# Antiaging agents: safe interventions to slow aging and healthy life span extension

**DOI:** 10.1007/s13659-022-00339-y

**Published:** 2022-05-09

**Authors:** Ji-Kai Liu

**Affiliations:** School of Pharmaceutical Sciences, South-Central University for Nationalities, Wuhan, 430074 People’s Republic of China

**Keywords:** Antiaging agents, Antiaging targets, Healthy lifespan extension, Natural products, Endogenous substances, Longevity

## Abstract

Human longevity has increased dramatically during the past century. More than 20% of the 9 billion population of the world will exceed the age of 60 in 2050. Since the last three decades, some interventions and many preclinical studies have been found to show slowing aging and increasing the healthy lifespan of organisms from yeast, flies, rodents to nonhuman primates. The interventions are classified into two groups: lifestyle modifications and pharmacological/genetic manipulations. Some genetic pathways have been characterized to have a specific role in controlling aging and lifespan. Thus, all genes in the pathways are potential antiaging targets. Currently, many antiaging compounds target the calorie-restriction mimetic, autophagy induction, and putative enhancement of cell regeneration, epigenetic modulation of gene activity such as inhibition of histone deacetylases and DNA methyltransferases, are under development. It appears evident that the exploration of new targets for these antiaging agents based on biogerontological research provides an incredible opportunity for the healthcare and pharmaceutical industries. The present review focus on the properties of slow aging and healthy life span extension of natural products from various biological resources, endogenous substances, drugs, and synthetic compounds, as well as the mechanisms of targets for antiaging evaluation. These bioactive compounds that could benefit healthy aging and the potential role of life span extension are discussed.

## Introduction

When we ask what is health? Under ideal conditions, health is people in daily life in a completely independent state of physical and mental activities. But in practice, health refers to a sufficiently independent physical and mental state in the activities of daily life. The health and survival of an organic organism is a dynamic equilibrium process between the process of damage and repair, occurrence and maintenance. The traditional concept of this property is called homeostasis. However, it does not contain dynamic processes and topics, including the network and complexity of biological systems’ interactions. Now, homeodynamics has replaced homeostasis, arguing that the internal environment of complicated biological systems is not permanently changeless, not in equilibrium, but the dynamic adjustment and interaction during the different levels of the organization. The “survival ability” or “buffering capacity” of a biological system ultimately determines the health of an individual. The ability to survive and maintain good health is known as the homeodynamic space. The three main features of homeodynamic space are damage control, stress response, and constant remodeling and adapting [1].

Aging is the gradual contraction of homeodynamic space. Biologically speaking, the survival of an organism is a continuous struggle between biochemical damage and repair described above. Many molecular, cellular, and biochemical pathways and their networks determine survival and lifespan [2, 3]. All age-related variations, such as decreased hormone levels and remodeling of immune function, may not be harmful, appear without any benefits, and they may be an adapting sign. Similarly, the results of stress can be beneficial or harmful, depending on the frequency, intensity, and duration of stress, the cost of energy consumption and other metabolic disorders. This understanding of aging has led to a shift in our approach to aging interventions from “anti-aging” to “healthy aging”. In order to achieve healthy aging, we must abandon disease-oriented research methods and adopt health-oriented prevention strategies [1].

Aging has traditionally been considered a “natural” and inevitable process. However, many people question this, arguing that aging is an unbeatable part of human nature rather than a modifiable risk factor. Over the past 30 years, several interventions and numerous preclinical studies have been shown to slow aging and increase the healthy lifespan of organisms from yeast, flies, rodents to non-human primates. Interventions can be divided into two broad categories: lifestyle changes and pharmaceutical/genetic regulation. The former includes caloric restriction and regular physical exercise. The latter includes an increasingly wide range of chemically unrelated molecules, including natural products, endogenous substances, approved drugs, and synthetic compounds. There is strong evidence that aging interventions will delay and prevent the onset of many chronic diseases in adults and older adults, and may safely and effectively extend the healthy lifespan of humans [4–6].

In the past 20 years, a few genetic pathways have been characterized that play a clear role in controlling the aging process and lifespan. Therefore, the genes in the pathways are attractive and potential anti-aging targets. Currently, many anti-aging drugs targeting the mechanisms of aging are being developed, including caloric restriction mimics, autophagy inducers, putative cell regeneration enhancers, DNA methyltransferase and histone deacetylase inhibitors as epigenetic regulators of gene activity [7]. Evidence on the overall effects of these compounds for health benefits remains limited, including epidemiological surveys searching the consequences of long-term exposure to these compounds on human health. Although not yet ready for human trials, all of this deserves additional research and special attention. The initial trial should be used first for the treatment of age-related diseases and conditions (not specifically aging). They should start from fewer subjects, apply for a relatively short period of time, and focus primarily on safety and tolerability. This approach can provide early clues to promising compounds for potential candidates and then deserve longer or more detailed aging studies [5].

Over the past century, average human life span has increased substantially. By 2050, 20% of the world’s 9 billion people are expected to be over 60 years old. It is clear that the exploration of new targets for these anti-aging agents based on biogerontology is a huge opportunity for the healthcare and pharmaceutical industries [7]. The present review would focus on the properties of slow aging and healthy life span extension of natural products from various biological resources, endogenous substances, drugs, and synthetic compounds as well as the mechanisms of targets for anti-aging evaluation, would discuss these bioactive compounds that could provide benefits in the aspect of healthy aging and potential role of life span extension.

## Natural products as antiaging agents

### Astaxanthin

Astaxanthin (**1**) is a kind of carotenoid that belongs to the subclass of xanthophyll. It is a nutrient with many clinical benefits and acts through unique cell membrane effects [8]. This compound quenches free radicals or neutralizes other oxidants by accepting or donating electrons without becoming a prooxidant or being damaged in the process. Its linear structure and polar-nonpolar-polar layout enable it to be inserted precisely into the membrane and across the entire width. The polar structure is the ionone ring, which has a strong ability to scavenge free radicals or neutralize other oxidants, mainly in aqueous environments, or also in the absence of water [9, 10]. The nonpolar intermediate segment acts as a series of conjugated carbon–carbon double bonds, giving the compound further oxidation resistance and the ability for the removal of high-energy electrons from free radicals and delocalization of their electron energy through carbon–carbon chains. At this location, astaxanthin can intercept active molecules in the hydrophobic interior and at the hydrophilic boundary of the membrane.

Astaxanthin has shown a variety of clinical benefits with good tolerability and safety. In double-blind, randomized controlled trials, it reduced oxidative stress by 5 mg or 20 mg/day in obese and overweight subjects and 5 mg, 20 mg or 40 mg/day in smokers [11, 12]. The results showed that oxidative DNA damage was inhibited, C-reactive protein and other inflammatory biomarkers were decreased, and tuberculin skin test immunity was enhanced [13, 14]. In another trial, daily astaxanthin doses of 6, 12, or 18 mg decreased triglycerides and increased HDL cholesterol and improved blood flow in microcirculation models [15]. In a small clinical trial, it improved cognition and promoted the differentiation and proliferation of neural stem cells in culture [16]. Astaxanthin improved vision and eye adaptation in several randomized controlled trials in Japan [17, 18]. In a double-blind, randomized controlled trials, the protective effect of astaxanthin on fertility and sperm function was evaluated. The researchers recruited 30 men from infertile couples, the female partner in subjects showed no obvious reason for infertility. They were randomly assigned to either astaxanthin group (16 mg a day) or a placebo group for 3 months. During this time, they were permitted to provide semen for intrauterine insemination and the pregnancies were recorded. After 3 months, sperm linear velocity was obviously increased in the astaxanthin group (P < 0.05) but not in the placebo group [19]. Sperm oxygen free radical production was significantly decreased in astaxanthin group (stimulated by the oxidant Phorbol ester) (P < 0.05). However, the pregnancy rate, a trial result that is most telling, reached 54.5% in the astaxanthin group compared to 10.5% in the placebo group (P < 0.05).

In a 2008 randomized controlled trial, the effect of astaxanthin on functional dyspepsia was evaluated [20]. One hundred and thirty patients aged 18–70 years with functional dyspepsia were selected for the clinical trial by detailed questionnaires, gastroscopy, and urea breath test for *Helicobacter pylori* 81 of the 130 patients (62%) tested positive for *H. pylori*. All patients were then randomly assigned to receive either 16 mg or 40 mg of astaxanthin daily or a placebo in a double-blind trial for 4 weeks. Gastrointestinal symptoms: abdominal pain, dyspepsia, and reflux were assessed by the rating scale (GSRS). The trial results were assessed by a standard questionnaire. Treatment lasted for 4 weeks, followed by follow-up after 4 weeks. There were no significant differences in the combined scores of abdominal pain, dyspepsia and reflux syndrome among the three groups. The same results were found at the end of the follow-up. The researchers concluded that astaxanthin had no effect on FD overall. However, the higher dose of astaxanthin (40 mg/day) did obviously reduce acid reflux-related symptoms compared to the 16 mg/day dose and placebo (P < 0.05) [20]. The high dose of astaxanthin also significantly improved well-being in the questionnaire for quality of life (P < 0.05).

The clinical success of astaxanthin goes beyond protection against oxidative stress and inflammation, and could demonstrate its promise to slow age-related decline. Astaxanthin cannot be produced in the human body and is mainly ingested through the diet, and almost all of the dietary intake containing astaxanthin comes from seafood. In nature astaxanthin is produced by algae, bacteria and fungi. Because these primary producers are often used as food, the substance is concentrated at the top of the food chain. It turns the flesh, skin or exoskeleton of animals such as shrimp, krill, crayfish, lobster, crab, salmon and trout into a natural intense red illumination. It is also fed as feed to farmed seafood for its coloring. Astaxanthin is also naturally present in the feathers of flamingos (which are the characteristic color of quails) and in their retinas [8]. Natural astaxanthin is mainly derived from commercially cultured single-celled algae *Haematococcus pluvialis* [21]. Synthetic astaxanthin is also emerging as an important product. Natural and synthetic astaxanthin differ in chemical composition, bioavailability, purity, or sensory quality. Natural astaxanthin is variable and exists as stereoisomers of 3S, 30S and 3R, 30R, and in free form or esterified forms. The algae *Haematococcus* primarily produces the 3S and 30S isomers, which are also present in the free form in wild Atlantic salmon. The yeast *Pfaffia rhodozyma* synthesizes mainly the 3R and 30R isomers, which are also occur in the esterified form in Antarctic krill. The vast majority of new dietary supplements approved by the FDA for natural astaxanthin use *H. pluvialis* extracts. Natural astaxanthin extracts usually contain other carotenoids (canthaxanthin, β-carotene, and lutein), which have related and similar biological activities depending on the source. Synthetic astaxanthin consists of a mixture of 3S, 30S, 3R, 30S, 3R and 30R isomers and may also contain traces of residual solvents and impurities [22].

Natural astaxanthin showed a good clinical safety profile, with no serious adverse side effects observed in any clinical studies, even at high doses (45 mg in 15 patients). In one clinical trial, at a dose of 30 mg, subjects were found to have red-colored stools and increased frequency of bowel movements. Its short-term dose of 100 mg/day and long-term daily doses averaging 8 to 12 mg have also not been reported to influence changes in human liver parameters [22].

### Curcumin

**Scheme 1:**
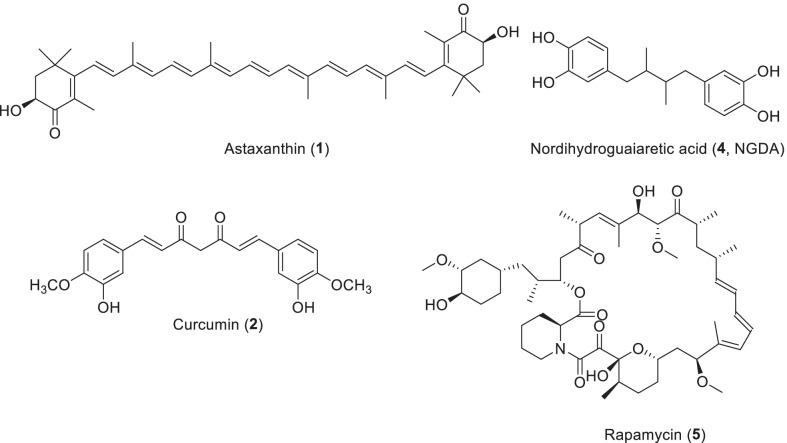
The structures of natural products as antiaging agents

*Curcuma longa* is a curry spice that originated in India. The rhizomes of *Zingiber officinale* (Zingiberaceae), ginger, as one of the most popular spices, has been planted in China for thousands of years for spices and medicine [23]. In recent decades, they have attracted great interest because they contain biologically active curcuminoids (curcumin, demethoxycurcumin, and bisdemethoxycurcumin). Curcumin (**2**) is a kind of natural yellow pigment, is very rare in natural diketone coloring matter, have strong tinting strength, bright color, strong heat stability, safety, non-toxic, can be widely applied in cakes, sweets, drinks, ice cream, wine, and other foods as a colorant, is considered to be one of the most valuable natural edible pigment (Scheme 1). At the same time, it is also the World Health Organization and the Food and Agriculture Organization of the United Nations regulation of the use of high safety of natural pigments. Its global market size is estimated at more than $500 million. Curcumin is a lipophilic plant polyphenol that has been shown to act as an anti-aging, anti-inflammatory, anti-cancer agent and antibiotic based on and in vivo studies and clinical trial results [24]. Curcumin comes in two tautomers, ketones—and enols. At both neutral and acidic pH, the ketone type dominates, while enol tautomer exists only in basic conditions. This can be explained by the formation of enol-type intramolecular hydrogen bonds [25].

Tetrahydrocurcumin was reported to extend the life span in a study for male mice [26]. It was confirmed that curcumin supplementation helps reduce some features of dementia in rodents. In adult mice, curcumin improved synaptic plasticity, neuronal repair, and hippocampal neurogenesis [27]. Another study showed that curcumin improved synaptic structure and quality in the hippocampal CA1 region of double transgenic mice [28]. Furthermore, curcumin can effectively reduce Aβ production in the double transgenic mice by inducing autophagy [29]. However, to date, no studies have evaluated the effects of curcumin on cognitive performance in humans.

There are over 15,000 publications on curcumin biological interactions, with about 50 published per week. A large number of published manuscripts on curcumin’s biological activity makes it almost impossible for researchers to follow the latest advances in this field. Recently, an excellent review discussed an in-depth evaluation of curcumin, a potential new direction for research [30]. The article provides a review of the basic medicinal chemistry of curcumin and demonstrates that curcumin is an unstable, reactive, poorly bioavailable compound and therefore not a lead compound, but they do not rule out the possibility that crude curcumin extracts may be beneficial to human health. The new study suggests that curcumin may affect the intestinal microflora, which is associated with a variety of chronic diseases. This hypothesis has not been fully tested, but may eventually provide a focal point for research into the therapeutic effects of curcumin.

### Morphine

Morphine (**3**) is a substance found naturally in poppies *Papaver somniferum*. Despite side effects of respiratory depression, tolerance, and addiction, it remains one of the most widely used painkillers in medical clinics. In addition, the presence of endogenous morphine in animals is widely accepted, including invertebrates and vertebrates.

The effects of morphine on the lifespan of fruit flies were studied. The 0.01, 0.05, and 0.25 mg/mL morphine hydrochloride (MH) were added into the medium, respectively, from the 5th or 54th day of adults. MH supplementation on the 5th day after birth obviously prolonged the average lifespan of male flies at all studied concentrations. The female fruit flies treated with only 0.25 mg/mL MH lived significantly longer than the control group [31]. Together with earlier studies in mice, [32] morphine supplementation was found to extend life span in vertebrates and invertebrates.

### Nordihydroguaiaretic acid (NDGA)

Nordihydroguaiaretic acid (**4**), isolated from the leaves of the Creosote shrub (*Larrea divaricata* or *Corillea tridentate*), an evergreen desert plant widely found in the southern United States and the northern border region of Mexico, is a natural lignan. Over the past century, intensive research has shown that nordihydroguaiaretic acid and its synthetic analogues have potential use in the treatment of diseases related to cancer, diabetes, bacterial and viral infections, and inflammation. Of particular note is the fact that terameprocol is in Phase I/II clinical trials as an anti-cancer agent. Tameprocol is a tetra-*O*-methyl derivative of nordihydroguaiaretic acid [33].

Nordihydroguaiaretic acid (NDGA) has anti-inflammatory and antioxidant properties. NGDA treatment (after 4 weeks of 2.5 g/kg NDGA diet) has been reported to extend the lifespan of genetically heterogeneous male mice, but not female mice [34]. NDGA was observed to increase survival in males but not females, which may be related to gender differences in homeostasis levels and/or drug metabolism. In previous studies, NDGA has also been reported to extend the lifespan of fruit flies, mosquitoes, and rats. There have been several studies on the effects of NDGA on brain aging. Treatment with NDGA extends life span, reduces oxidative stress, reduces neuroinflammation, and enhances memory in fly models of Alzheimer’s disease [35]. In mammalian models, NDGA prevents Aβ neuronal toxicity in vitro and reduces Aβ deposition and brain amyloidosis in transgenic mice with Alzheimer’s disease [36].

Nordihydroguaiaretic acid (NDGA) showed antioxidant and anti-inflammatory activities. After 4 weeks of administration with 2.5 mg of NGDA per gram of diet, NDGA was reported to be observed to prolong the life span and survival of male mice, but not females [34]. This may be related to gender differences as well as drug metabolism. In previous studies, NDGA has also been reported to extend lifespan in *Drosophila*, mosquitoes, and rats. There are also some studies on the effects of NDGA on brain aging. In a *Drosophila* model of Alzheimer's disease, treatment with NDGA extended lifespan, reduced oxidative stress and neuroinflammation, and enhanced memory [35]. In a mammalian model of Alzheimer’s disease, NDGA prevented Aβ neuronal toxicity and reduced Aβ deposition and brain amyloidosis in transgenic mice [36].

There is also evidence that chronic ingestion of nordihydroguaiaretic acid may cause hepatotoxicity in humans. Later, NDGA was discontinued as a food preservative when it was shown to be nephrotoxic in rats. NDGA has recently been found to be hepatotoxic to rats with an LD_50_ of 75 mg/kg, which was determined by raising serum alanine aminotransferase levels. Long-term use is associated with hepatotoxicity. In vitro studies showed that NDGA was biotransformed into an active intermediate in vivo, responsible for its toxicity [37].

### Rapamycin

Rapamycin (Scheme 1, **5**) is a macrolide isolated from *Streptomyces hygroscopicus*, an actinomycete from soil samples from Easter Island. Initially rapamycin was discovered as a new antifungal agent, and later it was found to show immunosuppressive and anticancer activity by inhibiting the mechanistic target of rapamycin (mTOR) [38]. mTOR is a serine/threonine protein kinase belonging to the PI3K phosphatidyl 3 kinase family that regulates not only growth and proliferation but also metabolism and aging. mTOR forms two distinct protein complexes, mTORC1 and mTORC2; the former is highly sensitive to rapamycin, whereas the latter is only chronically sensitive to rapamycin [39]. In the last decade, it was evident that genetic and pharmacological inhibition of mTORC1 prolongs lifespan and delays aging, but that inhibition of mTORC2 negatively affects mammalian health and lifespan and leads to many side effects of rapamycin [39].

In 2003, the TOR pathway was discovered as an important aging regulator in nematodes *Caenorhabditis elegans*, [40] and later in yeast *Saccharomyces cerevisiae*, [41] and fruit flies *Drosphila melanogaster* [42]. Rapamycin was first proposed as a potential anti-aging therapy.

One study showed that even when the treatment was started at a very old age, female mice lived a remarkable 14% longer, and male mice lived 9% longer [43]. Rapamycin supplementation increases median and maximum lifespan in male and female mice. Results from several independent research groups have shown that rapamycin extends the lifespan of different strains of mice. Beneficial effects have been reported for various treatment strategies, from age of onset (i.e., middle-aged, late-aged) to duration of treatment (i.e., transient, intermittent) to mode of administration (oral, intraperitoneal) [44, 45]. In mice, rapamycin supplementation improved several age-related indicators such as muscle strength, immune function, oral health, mitochondrial function, coordination and balance, pain perception, cardiovascular disease, and brain health [46, 47]. Many promising results support further research with rapamycin as a potential intervention for common neurodegenerative diseases associated with aging, including Alzheimer’s disease and Parkinson’s disease [48, 49]. A strict clinical trial is currently underway to evaluate the efficacy of a mild TORC1 inhibitor in Parkinson’s disease. Although many challenges remain in translating the remarkable efficacy of rapamycin therapy into routine clinical practice in age-related diseases, there is no doubt that the future of rapamycin therapy is bright [39].

### Resveratrol

**Scheme 2:**
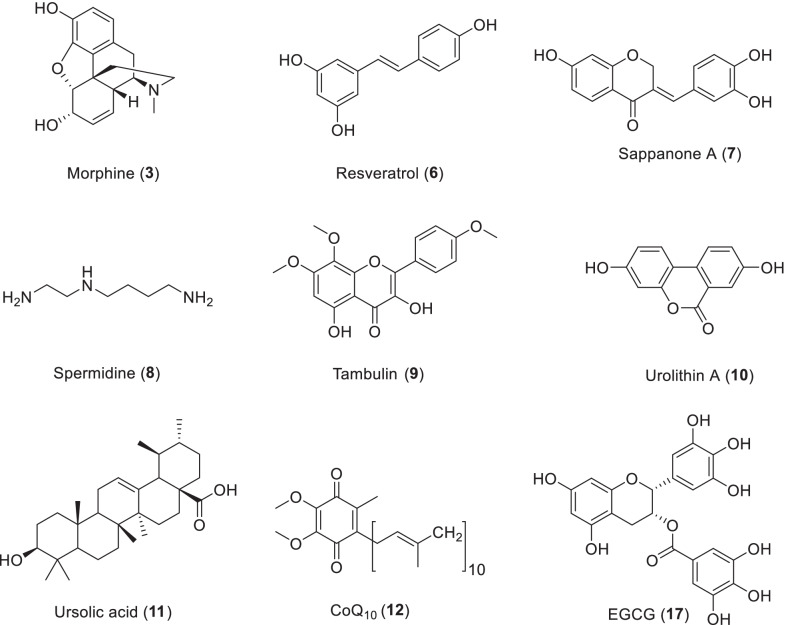
The structures of natural products as antiaging agents

**Scheme 3:**
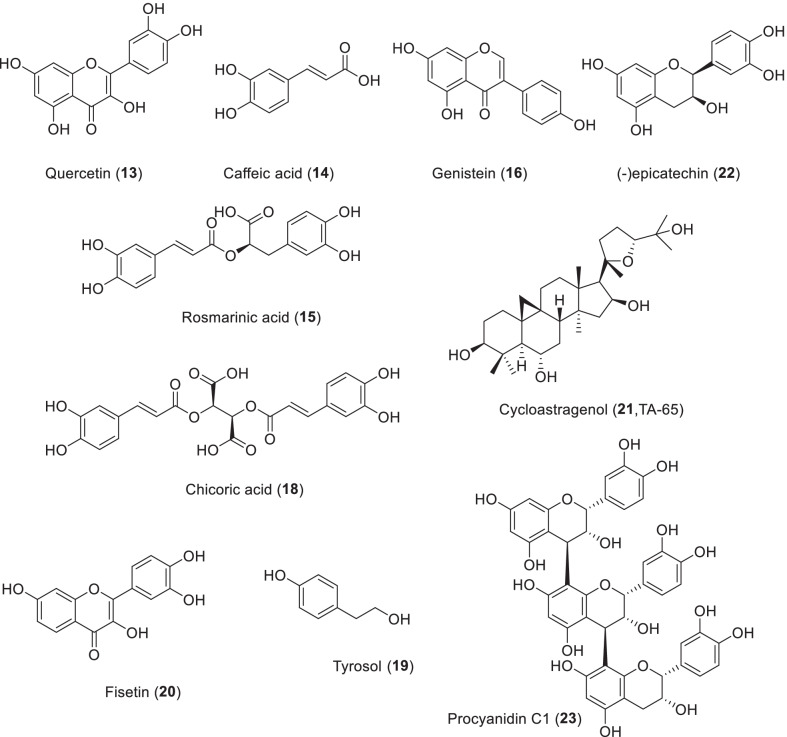
The structures of natural products as antiaging agents

Resveratrol (Scheme 2, 2,3,5,4ʹ-trihydroxytrans-stilbene, **6**) is a compound of the stilbene family that is a naturally occurring plant polyphenol and a phytoalexin, which was produced when plants are injured or attacked by pathogens. Resveratrol is mainly found in foods such as grape skins, blueberries, raspberries and mulberries. The interest in resveratrol began with the so-called “French paradox”, in which the French population has a relatively low incidence of cardiovascular events despite eating a diet high in saturated fats. The cardiovascular health benefits of red wine consumption are due to its high concentration of the non-flavonoid resveratrol [50] However, after Jang et al. [51] published in Science, clarified the chemical protective effect of resveratrol, and subsequently reported that it activates sirtuin deacetylase and prolongs the life span of yeast, [52] researches on the effects and properties of this compound began to grow exponentially. Since then, many in vitro and in vivo experimental animal studies have found varying degrees of evidence that resveratrol exerts health benefits in a variety of diseases, including cancer, cardiovascular disease, eye disease, and neurodegenerative disorders. Resveratrol can act in a variety of biological pathways through different mechanisms of action, including oxidative stress, inflammation, mitochondrial dysfunction, apoptosis, promotion of survival or angiogenesis. Oxidative stress and inflammation play a key role in the onset and progression of age-related eye diseases such as glaucoma, cataracts, diabetic retinopathy and macular degeneration, leading to progressive loss of vision and blindness [50].

A recent review discussed the positive effects of resveratrol on longevity, age-related disease, and health maintenance [53]. In yeast, resveratrol extends the lifespan of *Saccharomyces cerevisiae* by 70% through direct activation of SIR2 activity [54]. Similarly, resveratrol extends the lifespan of nematodes by stimulating SIR-2. Resveratrol’s life-extending effect on *Drosophila melanogaster* seems to depend on gender and dietary nutrients. Resveratrol extended the life span of *Apis mellifera* by 33–38% on any diet [55]. It was reported that resveratrol supplementation extended lifespan and delayed age-related motor and cognitive decline in *N. furzeri* [56]. In contrast, resveratrol supplementation had no effect on *Daphnia* [57]. Resveratrol improved vascular health without extending the lifespan of mice. While no studies have shown that resveratrol extends life span in healthy mammals [58]. Potential therapeutic effects of resveratrol in improving ovarian function have also been reported; however, since it also has anti-decidual effects in the endometrium. Moreover, since its teratogenic potential has not been ruled out, resveratrol should be avoided during the luteal phase and during pregnancy [59].

The bioavailability of resveratrol showed a high degree of inter-individual variation, not related to age or gender. Despite the low bioavailability of resveratrol, it still acts in vivo. This may be due to the reconversion of its metabolites from the sulfate and glucuronide of parent resveratrol to resveratrol in target organs, such as the liver. Based on different animal and human studies, nearly 20 resveratrol-derived metabolites have been detected in plasma, urine and some tissues. Among these metabolites are trans- and/or cis-mono and disulfates, monoglucuronides and diglucuronides from parent resveratrol, as well as equivalent conjugates of the microflora-derived metabolite dihydroresveratrol (DHRES). Another possible explanation is entero-liver recycling of resveratrol metabolites, which are subsequently broken down and reabsorbed in the small intestine. Based on investigations of the health-related effects of stilbene, the metabolism by human gut microbiota shows significant inter-individual differences. Finally, effects in vivo can be explained by its metabolites’ activity [60].

For years, the prevailing view was that increasing resveratrol intake led to better clearance of reactive oxygen species (ROS) and, therefore, to cytoprotective effects. However, under certain conditions, antioxidants may act as pro-oxidants, causing accelerated lipid peroxidation and/or inducing DNA damage. Some studies reported the in vivo toxicity of resveratrol in rats. For example, high doses of resveratrol can cause inflammation of the heart, dilatation of renal tubules, papillary necrosis, acute inflammation of the pelvic area, and death from severe kidney disease. In addition, high doses of resveratrol could cause significant increases in blood urea nitrogen, creatinine levels, and liver enzymes. Resveratrol’s role in renal fibrosis in mice has also been studied. A low dose of resveratrol (≤ 25 mg/kg) can partially improve renal function in mice with unilateral ureteral obstruction. The anti-fibrosis effect of high dose resveratrol (≥ 50 mg/kg) disappeared, but aggravated renal fibrosis. The biological effects of resveratrol, as well as the results in vitro and in vivo, are closely associated to the hormetic effect, with low doses of resveratrol generally associated with beneficial effects and high doses usually having toxic effects [61].

Although resveratrol has a variety of pharmacological activities, how it leads to different or even completely opposite effects in different diseases remains unclear. In addition, many of resveratrol’s effects and mechanisms in humans have not been confirmed. Poor bioavailability of resveratrol is another major drawback of this molecule. The poor bioavailability of this molecule is another of major disadvantage of resveratrol. There is a lack of data on human studies, and comprehensive randomized clinical trials must be conducted on resveratrol’s preventive potential, effectiveness and safety in different diseases before such claims can be confirmed [50].

### Sappanone A

Sappanone A (**7**) is an isoflavone isolated from *Caesalpinia sappan*. It selectively binds to inosine monophosphate dehydrogenase 2, a protein involved in aging. It has been reported that the life-prolonging and health care effect of sappanone A in *Caenorhabditis elegans* and its potential pharmacological mechanism were investigated. The larvae were treated with 0–50 μM sappanone A to observe the effects of sappanone A on the longevity of the larvae, and the health status of the larvae was evaluated by measuring the locomotor capacity, feeding capacity, reproductive capacity, heat resistance, lipofuscin content, ROS accumulation, and other indicators. The transcription of insulin/insulin-like growth factor-1 signaling pathway and heat stress response gene was examined by RT-QPCR to explore the possible mechanism. In addition, the subcellular distribution of GFP labeled DAF-16 was detected, and the interaction between sappanone A and HSP-90 protein was mimicked by molecular docking. It was found that sappanone A can extend the life span of *C. elegans* and enhance their motility and heat resistance. Feeding and breeding are not affected. ROS and lipofuscin accumulation decreased. Mechanism studies showed that DAF-16 and HSP-90 gene expression levels were up-regulated. Moreover, DAF-16 is easily located in the nucleus. In the simulation, sappanone A was docked into the active pocket of the HSP-90. Sappanone A (50 μM) can prolong the longevity of *C. elegans* and slow down senescence by adjusting the IIS pathway. DAF-16 is particularly crucial in longevity regulation. HSP-90 is joined to enhance the heat tolerance [62].

### Spermidine

Spermidine (**8**) is the most abundant polyamine in different human tissues, and the concentration of intracellular spermidine decreases during the natural aging process of the body [63]. Sperm contains high concentrations of spermidine, which prevents cellular senescence and allows long-term survival of the germ cell line. The homeostasis of spermidine is influenced by nutritional intake, intestinal microflora, endogenous biosynthesis, degradation and intercellular transport systems [64]. High levels of spermidine are found in a wide range of foods, such as fresh peppers, wheat germ, broccoli, cauliflower, and cheeses, while higher levels are found in soy products such as natto, shiitake, and durian [65].

As a mimetic of caloric restriction, spermidine has significant cardioprotective and neuroprotective effects and activates anti-tumor immunosurveillance in rodent models. In addition, polyamines in diet have been associated with reduced cancer-related and cardiovascular mortality in human epidemiological surveys. Spermidine maintains mitochondrial function, shows anti-inflammatory activities, and prevents stem cells from aging. Mechanistically, it follows the same biochemical pathway as other caloric restriction mimics: it induces protein deacetylation and relies on autophagy [66].

Administration of spermidine increased the survival of yeast, nematodes, fruit flies and human immune cells and reduced age-related mortality in mice. In aging flies, spermidine treatment improved memory, an effect associated with increased autophagy in neuronal tissue [67]. In rats, spermidine treatment (5 and 10 mg/kg) improved motor performance and reduced pro-inflammatory cytokines and oxidative stress in the striatum [68]. In a rodent model of Huntington’s disease, injection of spermidine into the striatum improved recognition memory for novel objects [69]. Several studies have been conducted on spermidine treatment in humans. Long-term spermidine supplementation (1.2 mg/day) is safe and well tolerated in the elderly [70]. Food questionnaires have shown that high dietary intake of spermidine lowers blood pressure and benefits cardiovascular disease, while spermidine supplementation (1.2 mg/day for 3 months) improves memory performance in older adults (60–80 years) with subjective cognitive decline [71].

As noted above, spermidine administration increases survival in animal models. A study reportedly aimed to test a potential link between dietary spermidine levels and mortality in humans. Their findings provide epidemiological support for the concept of spermidine-rich nutrition and improved human survival [64].

### Tambulin

Using the *Caenorhabditis elegans* model system, the effects of tambulin, a hydroxyl substituted flavanol (3, 5-di hydroxy-7, 8-dimethoxy-2 -(4-methoxy-phenyl) chromen4-one, **9**) isolated from *Zanthoxylum armatum* fruits on longevity and neuroregulatory activity were studied. The results showed that tambulin significantly increased the longevity and stress tolerance of worms, while alleviating the effects of lipofuscin and protein carbonyl on aging biomarkers. Consistent with reduced ROS levels, mRNA expressions of ROS-scavenging genes SOD-1, SOD-3 and CTL-2 were up-regulated after tambulin treatment. Upregulation of DAF-16 suggests that insulin signaling is involved in tambulin mediated longevity. Tambulin therapy can reduce the presentation of Parkinson’s disease (PD) by reducing the level of α-synuclein, lipid accumulation, and improving motor behavior and dopamine levels. In conclusion, the relief of PD symptoms mediated by tambulin may be related to the anti-protective mechanism of PD, mainly through up-regulation of mRNA expressions of lagr-1, ymel-1, pdr-1, ubc-12 and lrk-1. Tambulin represents a potential molecule for anti-aging and Parkinson’s disease [72].

### Urolithins

Urolithins are a subgroup of 3, 4-benzo coumarin, isolated from the beaver odor gland. They are found in many microorganisms, plants, human feces and animal feces. In the last decade, research interest in urolithins has increased considerably. The results of studies to date confirm that urolithins can regulate oxidative stress and ameliorate tissue damage through different mechanisms [73]. Recent reports have shown that urolithin A (**10**) significantly increases type I collagen expression and decreases matrix metalloproteinase 1 expression. After 20 and 50 μM urolithin A treatment, the expression of collagen mRNA was obviously increased, and the expression of MMP1 mRNA was obviously decreased in a dose-dependent manner [74].

Urolithins are intestinal metabolites produced by foods rich in ellagitannin such as blackberries, strawberries, pomegranate, tea, walnuts, raspberries, and raspberries. Urolithins have a variety of biological activities, including cardiovascular protection, anti-inflammatory, anti-cancer, anti-diabetic and anti-aging, and have attracted increasing interest in recent years. The results of many in vitro and in vivo based experimental studies support the beneficial effects of urolithins in the treatment of diseases such as Alzheimer’s disease, type 2 diabetes, liver disease, cardiovascular disease and various cancers. Urolithins are involved in a variety of cellular mechanisms, including inhibition of MDM2-p53 interaction, regulation of mitogen-activated protein kinase pathways, and inhibition of NF-κB activity. Anti-aging activity is the most intriguing and perhaps the most important property of urolithin A, which has been intensively studied in recent years due to its unique role in activating mitochondrial autophagy and mitochondrial biogenesis. A recent clinical trial has shown that a dose of up to 2500 mg/day of urolithin A is safe and improves mitochondrial biomarkers in elderly patients. Given the importance of mitochondria in the pathophysiology of many diseases, urolithins deserve further in-depth study, especially in clinical trials, to reveal its additional clinical significance. In addition to the nutritional value of urolithin, recent studies have shown that urolithins can be used as medicines to prevent or treat various diseases. It was reviewed that the potential role of urolithins as novel therapeutic agents, with a special focus on the signaling pathways underlying its biological effects [75].

### Ursolic acid

Ursolic acid (UA, **11**) is a usual pentacyclic triterpenoid acid found in apples, prunes, and papaya, as well as in some herbs, and located mainly in waxy coatings, leaves, and bark. UA induces autophagy in a multicellular culture model and in vivo [76, 77]. Both in vitro and rodents in vivo studies have shown that UA has anticatabolic activity, counteracts age-related deficiencies in skeletal muscle strength, and quality, and promotes muscle fiber regeneration [78]. These results support the view that UA can delay aging by increasing the levels of SIRT1 and SIRT6. UA delays or ameliorates aging by boosting anti-aging biomarkers in the hypothalamus. In addition, UA provides suitable conditions for mitochondrial biogenesis and function by promoting PGC-1b overexpression. An aging animal model (C57BL/6) was used. UA was dissolved in corn oil (20 mg/mL) and injected (200 mg/kg I.P injection) to mice twice a day for 7 days. After several treatments, the hypothalamus was isolated from the mice, and the tissue prepared was observed by immunofluorescence microscopy. UA significantly increased the overexpression of SIRT1 (~ 3.5 times) and SIRT-6 (~ 1.5 times). In addition, UA substantially increased the protein levels of a-Klotho and PGC-1b. The protein is associated with the metabolism of phosphate, calcium, and vitamin D [79].

UA leads to the activation of AMPK by reducing cellular energy states (ADP and ATP), which causes the up-regulation of SIRT1 expression and thus regulates the transcription of PGC-1α. PGC-1α can promote oxidative metabolism and glycolysis into oxidized fibers, and also promote myoglobin expression and mitochondrial biogenesis. On the other hand, UA promotes myoblast proliferation by increasing SIRT1 expression in satellite cells [80]. The longevity extension effect of UA was demonstrated in the nematode model *Caenorhabditis elegans* [81]. In WT animals, UA regulates the lifespan of nematodes in a diets-restriction-dependent manner and inhibits the accumulation of toxic proteins by inducing JNK-1 protein aggregation [82]. The effects of UA on longevity, motor activity and health, including fecundity, intestinal integrity and microbiota composition, of *Drosophila melanogaster* strain W^1118^ were investigated. Phenotypic and molecular target studies showed that UA intake obviously prolonged the lifespan and healthy lifespan of male flies, while female fruit flies did not benefit from UA intake. Current data suggest that UA regulates its ability to promote health by upregulating srl expression, which may also be due to the regulation of flies’ microbiota [83].

### Coenzyme Q10

Coenzyme Q10 **(12**) is a fat-soluble substance that is directly involved in the body’s energy production system and plays an important role in the mitochondrial electron transport system. In addition, it has recognized roles in antioxidant and genetic induction. Coenzyme Q10 was first synthesized in 1958 and was approved for the treatment of congestive heart failure in Japan in 1974. Since then, a lot of research has been conducted worldwide on the function and action of coenzyme Q10. The data confirmed that the compound is safe and effective. In the United States, coenzyme Q10 has been sold as a dietary supplement under the Dietary Supplement Health and Education Act since 1994 [84]. As this drug has been promoted in clinical areas such as Parkinson’s disease, attention has been focused on improving its bioavailability and safety. In Parkinson’s disease, a daily dose of 1200 mg of coenzyme Q10 can be effective in slowing the progression of the disease [85]. When coenzyme Q10 is taken with meals, intestinal absorption does not exceed 3% of the dose given; In addition, the enrichment of coenzyme Q10 in functional foods is very limited due to its lipophilic properties [86].

Single administration studies in rats and humans have shown that P40 (water-soluble coenzyme Q10 powder, its content 40 W/W%) absorbs better than classical oil formulations. Long-term administration studies confirmed that this improved bioavailability led to an obvious increase in plasma [84]. In order to improve the bioavailability of coenzyme Q10, several advances have been made by reducing particle size, enhancing solubility and using new drug carriers [87]. Solubility can be improved through solid dispersion, prodrug, complexation, ionization, etc. New drug carriers such as liposomes, microspheres, nano-emulsions, nanoparticles and self-emulsifying systems.

In a randomized, double-blind, placebo-controlled study, 3 months of administration of a combination of water-soluble coenzyme Q10 (50 mg) and fish collagen (4.0 g), exhibited some beneficial effects on the skin as it increased dermal density and reduced periorbital wrinkle, improved skin smoothness [88]. Coenzyme Q10, taken orally in the form of Ubisol-Q10, is effective in preventing the progression of neurodegeneration at concentrations much lower than other oil-soluble formulations previously tested and a dose within 12 mg/kg/day [89]. Other clinical trials have demonstrated the role of water-soluble CoQ10 preparation in the prevention of age-related deafness [90, 91]. Recently, the beneficial role of Coenzyme Q10 in aging was reviewed [92, 93].

### Others (vitamins A and E, quercetin, caffeic acid, rosmarinic acid, genistein, EGCG, Protandim, chicoric acid, tyrosol, fisetin, TA-65, procyanidins)

Based on current studies in humans and animals, there are evidence that vitamins A and E may be beneficial for longevity only in the early stages of life, inferring that the production of these benefits may also depend on the presence of an optimal concentration level of them. To date their mechanisms have not been well established [94].

Quercetin (**13**), caffeic acid (**14**) and rosmarinic acid (**15**) can induce hormetic dose-responsive to prolong life span in *Caenorhabditis elegans*. The three polyphenols showed different antioxidant properties in vivo, indicating different modes of action (Scheme 3). In addition, the *qsr-1/unc-43/sek-1* pathway and *sir-2.1* were found to be common genetic participants in caffeic acid and rosmarinic acid involved lifespan extension in worms. *C. elegans* is apparently able to recognize these polyphenols and activate immune response pathways. They do not lead to an indirect dietary restriction effect that prolongs life, but they do cause substantial energy redistribution. These findings highlight the diverse actions of polyphenols in vivo and demonstrate that they can target aging process in whole life, not just improve survival in old age [95]. Quercetin (300 μM) was found to have a moderate effect on growth, while DMSO (as a control) had no effect on longevity or growth rate. In *Podospora anserina* WT treatment, the mean life expectancy increased by 10.2% and the maximum lifespan increased by 16.6% [96].

In normal culture, the presence of genistein (**16**) significantly extended the longevity of nematodes. Furthermore, genistein enhances the survival rate of nematodes under thermal and oxidative stress conditions. Further studies exhibited that the enhanced stress resistance of *C. elegans* mediated by genistein may be related to the enhanced expression of superoxide dismutase (SOD-3) and heat shock protein (HSP-16.2), which are common stress resistance proteins. In addition, no significant changes in aging-related factors induced by genistein were observed. The usual aging-related factors includes reproduction, food intake, and growth. It suggests that the life span extension activity of genistein was not associated with these factors. Genistein can also up-regulate the motility of aged nematodes, indicating that genistein can affect the healthspan and longevity of nematodes. The results showed that genistein had beneficial effects on the longevity of nematodes under normal and stress conditions by increasing the expression of anti-stress proteins [97]. However, the evidence was given for a new biotransformation pathway in *C. elegans* that leads to the derivatives formation of glucoside and glucoside phosphate, which so far have not been found in mammals. Therefore, there are huge differences in the metabolism of genistein in mammals and nematodes, which may have a great influence on their biological activity. The differences need to be properly considered when *C. elegans* is used as a model to evaluate possible health or aging effects [98].

Green tea has been found to prolong the life span of different animal models. In *C. elegans*, daily administration of 220 μM EGCG (**17**) increased life expectancy by 14% [99]. In *Drosophila melanogaster*, the average lifespan of male flies fed on green tea extract increased by 16%, and superoxide dismutase (SOD) and catalase were correspondingly upregulated [100]. In male mice, it was found that green tea administrating mice lived longer than mice of control group. EGCG significantly extended the median life span of the rats to 105 weeks. One study reported that EGCG delayed death in healthy rats and reduced its age-related oxidative stress, inflammation, and liver and kidney damage [101]. Further studies showed that the significant life span extending effects of EGCG on nematodes could be attributed to its free radical scavenging effect in vitro and in vivo and its upregulation of SOD-3 and HSP-16.2 [103]. The gender-specific effect of green tea on the life span of *Drosophila melanogaster* was studied and it was reported that green tea could only prolong the life span of male flies. This effect was found to be independent of typical aging interventions such as calorie restriction, regulation of oxidative energy metabolism and increased tolerance to stress. The results suggest that green tea prolongs the longevity of male fruit flies by reducing their reproductive potential, possibly by restricting iron intake [102]. The average life expectancy of EGCG increased by 13.1%, 8.0% and 11.8% at 0.1–10.0 μg/mL, respectively, under 35 °C high temperature stress. Under oxidative stress, EGCG increased the average lifespan of *nematodes* by 172.9%, 177.7% and 88.5% at 0.1–10.0 μg/mL, respectively. EGCG could not prolong the longevity of nematodes under common culture conditions. Further studies showed that EGCG had a obvious life span extension effect on nematodes, which may be associated with the activities of scavenging free radicals and up-regulating stress-resistance related proteins such as superoxide dismutase-3 and heat shock protein − 16.2 [103].

Protandim (Prot) is a mixture of five plant extracts. The five extracts contain bacosides, curcumin, epigallocatechin-3-gallate, silymarin, and withaferin A. Components consisting of low concentrations of each compound are used to activate Nrf2/ARE, by design principle, providing strong, synergistic effects and to minimize off-target side effects [104]. Studies have shown a 30% increase in superoxide dismutase and a 54% increase in catalase in healthy subjects who took Prot orally for more than 3 months [105]. Biochemical and histological surveys in mice showed that Prot administration inhibited oxidative stress, cell proliferation, and tumor promoter-induced inflammation. In addition, Prot treatment has been reported to protect the heart from oxidative stress and fibrosis in a rodent model of pulmonary arterial hypertension. In a follow-up study, only male mice were found to show a positive effect of Prot in life span extension. This gender-induced difference may be attributed to males and females metabolizing and/or eliminating the five compounds in the mixture in a different way. While the median lifespan of male mice in the current study was significantly increased, they did not show a maximum lifespan extension, suggesting that Prot’s effects are most beneficial early in life [106].

Another compound, chicory acid (**18**, CA), has been found to increase insulin secretion and glucose uptake in INS-1 and L6 cell lines, respectively. CA induced apoptosis of 3T3-L1 preadipocytes through ROS-mediated phosphatidylinoside 3 kinase/protein kinase B and mitogen-activated protein kinase signaling pathways, a mechanism that may explain the role of chicoric acid containing plants in obesity traditionally used. Concentration-dependent longevity was observed in CA. The data suggest that CA is a potent antioxidant that activates the AMPK pathway in the L6 myotubes. Similar to other AMPK activators, CA extends the lifespan of nematodes, an effect that can be measured even in the micromolar range [107].

Tyrosol (**19**) is a lipid-soluble phenol isolated from olive leaves and extra virgin olive oil. The results show that important molecular mechanisms directly associated with longevity are affected by tyrosol treatment in nematodes, suggesting that this molecule may play an important role in conserved genetic pathways to extend the longevity of the entire organism [108].

Fisetin (**20**) is a bioactive flavonol widely found in vegetables and fruits such as strawberries, apples, persimmons, cucumbers, and onions, with the highest concentration in strawberries (160 g/g). Fisetin is a highly fat soluble compound that easily crosses cell membranes and is assembled inside cells. This allows fisetin to have diverse beneficial effects, including anti-inflammatory, neuroprotective, anticancer and anti-diabetic effects. Fisetin was found to be a promising analogue of caloric restriction, effectively maintaining redox homeostasis during rat erythrocyte senescence. In addition, fisetin administration effectively reduces the excretion process associated with aging [109]. Fisetin is a potent compound in reducing aging markers in mouse embryonic fibroblast senescence induced by oxidative stress and human fibroblast senescence induced by the genotoxin etoposide. Fisetin also decreased markers of aging in aged mice, and in human adipose tissue explants. Fisetin treatment increased health and longevity in WT mice and aged mice [110]. It inhibits a variety of signal kinases, including the PI3K/mTOR, and is perceived as a natural dual inhibitor for this pathway. Senolytics (fisetin and dasatinib plus quercetin) have been shown to extend the lives of mice [111].

Telomere activator-65 (TA-65), also known as cycloastragenol **21**, is a potent small-molecule telomerase activator with the ability to extend telomeres length, which is purified from the plant *Astragalus membranaceus* [112]. Since 2007, it has been commercially marketed as a nutritional product that increases the average telomere length of haploinsufficient mouse embryonic fibroblasts (MEFs) and reduces the percentage of very short telomeres and DNA damage [113]. In a 1-year double-blind clinical trial, 38 patients were randomized into two groups, one with oral TA-65 for intervention and the other with placebo as a control. The macular function was measured by micro-perimetry as the primary index. Compared with the control group, oral TA significantly improved the macular function of patients [114].

Apple polyphenols are mainly composed of procyanidins (PC), consisting of (−)-epicatechin (**22**) and (+)-catechin. In order to study the anti-aging effect of PC on *C. elegans*, *C. elegans* was treated with PC. Treatment with 65 µg/mL PC extended the average life span of wild-type N2 and FEM1 nematodes by 12.1% and 8.4%, respectively, similar to resveratrol. In addition, 100 µg/mL AP significantly increased the average life span of the same worms by 12.0% and 5.3%, respectively, similar to PC. PC treatment had no effect on the longevity of sir-2.1 worms, which lack the activity of the protein deacetylase that is dependent on NAD^+^, a member of the sirtuin family. These results suggest that PC has a sir-2.1-dependent anti-aging effect on *C. elegans*.

Apple polyphenols contain mainly procyanidins (PC) composed of (−)-epicatechin (**22**) and (+)-catechin. To investigate the anti-aging effect of PC on nematodes, treatment of *C. elegans* with 65 µg/mL PC increased the mean lifespan of wild-type N2 and FEM1 nematodes by 12.1% and 8.4%, respectively, with activity comparable to that of resveratrol. In addition, similar to PC, 100 µg/mL apple polyphenols significantly increased the mean lifespan of these nematodes by 12.0% and 5.3%, respectively. PC treatment had no effect on the lifespan of sir-2.1 nematodes, which lack NAD^+^-dependent protein deacetylase activity [115]. Proanthocyanidin C1 (**23**, PCC1) from grape seed extract was recently found to improve the healthy life span and longevity of mice by its effect on senescent cells. Through screening the natural product library, it was found that PCC1 had a specific effect on senescent cells. At higher concentrations, PCC1 may selectively kill senescent cells by promoting ROS production and mitochondrial dysfunction. In mice, PCC1 consumes senescent cells in the therapeutic injury tumor microenvironment and improves therapeutic effects when used in combination with chemotherapy. Intermittent PCC1 intake, senescent cell implantation or naturally aged mice can improve physical function and prolong survival. PCC1 is considered as a natural senotherapeutic agent which exhibits activity in vivo. It has great potential to be further developed as an effective intervention in clinics to delay, reduce or prevent age-related pathology [116].

## Endogenous substances and life span extension

### Alpha-ketoglutarate and Oxaloacetic acid

α-Ketoglutarate (**24**, 2-oxypentanedioic acid or 2-oxy-glutaric acid) is present in all organisms and is an important small molecule metabolite in cells. α-Ketoglutarate (AKG) is an intermediate in the tricarboxylic acid cycle (TCA). As an anion of ketoglutaric acid AKG is also involved in the metabolism of nitrogen and amino acid. Numerous studies have demonstrated that intermediates of the TCA cycle show other important physiological functions in addition to their metabolic roles. They can be considered as gene expression effectors, signaling molecules, and stress response regulators [117, 118]. Although AKG is an intracellular metabolite, it is also present in blood plasma. Plasma levels of AKG were found to be obviously lower in older adults compared to younger adults—from 1 mg/mL to a few ng/mL. Given this fact, and given the diversity of AKG physiological functions, reduced endogenous AKG levels may be a marker of aging. Several studies have reported longevity effects of AKG supplementation on *C. elegans* and *D. melanogaster* because it inhibits TOR kinase, acting on similar effects to that induced by dietary restriction [119, 120]. AKG supplementation has also been found to prolong longevity in yeast and mice [121, 122]. It is believed that aging is associated with dysfunction of the TCA cycle, leading to changes in the proportion of intermediates products in the TCA cycle. This, in turn, may cause changes in DNA methylation that initiate aging. AKG-dependent DNA and histone demethylases are part of epigenetic mechanisms that act in the control of aging [123]. In addition, AKG, as an antioxidant, may help to reduce the expression of age-related oxidative stress, alleviate the adverse effects of stressors through ROS, and simulate dietary restriction.

Oxaloacetate (**25**) is an intermediate in the citric acid cycle. It is very important for regulating energy and maintaining glucose homeostasis. In *C. elegans* model, it has been involved in a nutrition-sensing pathway through a mechanism involving FOXO and AMP-activated protein kinases [124].

### Dehydroepiandrosterone (DHEA) and 17α-estradiol

Dehydroepiandrosterone (DHEA, **26**) is an endogenous steroid hormone secreted by the reticulum zone of the adrenal cortex in a pattern associated with age. It is present in plasma in higher concentrations than any other steroid hormone. Adrenal secretion of dehydroepiandrosterone (DHEA) begins at puberty, peaks at the age of 20, and then declines with age. Extracts of wild yams, especially Mexican yams, can be processed in the lab to produce DHEA. Yam is a variety of Dioscoreaceae that helps isolate diosgenin, which is converted to steroids including dehydroepiandrosterone (DHEA). The fact is that DHEA plays several important roles in the aging process, but its major biological functions have not been explored. But by age 80, blood levels have dropped 95%, to about 10 to 20% of what they were at age 20. DHEA is known to extend the lifespan of rodents by 50%, making them look younger. Animals taking DHEA supplements retained glossy black hair, while others turned gray in the process [125].

17α-estradiol (**27**) is an endogenous steroid that binds with the estrogen receptor. It is about 200 times less active as a hormone than its optical isomer, 17β-estradiol. 17α-estradiol decreases inflammation, enhances mitochondrial health, maintains metabolic health, and alleviates oxidative damage in aging. Mice treated with 17α-estradiol (initiating at 10 months of age with 14.4 ppm in the diet) had a 19% increase in life expectancy in males (but not in females), which was based on enhanced glucose tolerance and insulin sensitivity [106]. It has been put forward that 17α-estradiol supplementation can prevent neurodegeneration during aging due to the abundance of estrogen receptors in neural tissue [126].

### S-Linolenoyl glutathione

Glutathione (GSH) is a non-protein thiol that is widely present in mammalian cells and functions as an endogenous free radical scavenging mechanism, as a major reducing agent that acts as an antioxidant by strictly controlling the redox state. The effects of glutathione shortage in many pathologies have notified some researchers to develop novel alternative strategies to maintain or restore glutathione levels in these patients. At present, the half-life of glutathione is short, and the plasma membrane permeability of glutathione is low. These limit its application as a therapeutic agent. Therefore, a great deal of work has concentrated on developing carriers and conjugates which allow cellular uptake of glutathione, showing particular promise with prodrugs, mimics, and analogues. The results showed that LIN-SG thioester (**28**) supplementation in diet characteristically increased the lifespan of C-terminal glycine ethyl ester of GSH, vitamin E and linolenic acid of wild-type N2 nematode strain by increasing the reduction capacity of GSH in cells. Therefore, lin-SG derivatives can protect nematodes from paralysis and oxidative stress caused by Aβ/H_2_O_2_ exposure. Studies have shown that proper intake of lin-SG can promote the up-regulation of SIR-2.1 in N2 nematodes [127].

### Melatonin

Melatonin (**29**) is a neurohormone produced by pineal gland at night. Because of its critical role in adjusting sleep–wake cycles and circadian function, it leads to the wide adoption of primary treatment for sleep disorders and some other conditions. It is evident that melatonin is involved in aging-related processes because we observed that loss of melatonin secretion and decrease in circadian melatonin rhythm amplitude are closely related to different age-related pathological conditions. Its potential to prolong longevity has been repeatedly described in fruit flies and mice [128–130].

Many studies have shown that it can act as a potent antioxidant that, through its amphiphilic properties, allows it to cross physiological barriers, such as the blood–brain barrier, thereby reducing the level of oxidative damage in the aqueous and lipid cell environments. It also characteristically affected the expression of enzymes superoxide dismutase, glutathione peroxidase and catalase, and influenced antioxidant activity. In addition, melatonin’s anti-aging effects may be associated with its ability to modulate inflammation and immune responses by activating or inhibiting these processes. The therapeutic effects are thought to be associated with its ability to regulate the nuclear factor erythrocyte 2-associated factor 2, serving as a sensor of oxidative stress, and playing anti-inflammatory roles via antioxidant responses. One of melatonin’s more important abilities in anti-aging research is its potential to treat age-related neurodegenerative diseases such as Alzheimer’s and Parkinson’s. Importantly, the molecular pathways of melatonin action include insulin/IGF-1 pathway, phosphatidylinositol-3-kinase-protein kinase B/Akt pathway, mTOR pathway, SIRT1 and other molecular pathways related to autophagy and energy metabolism regulation. All these pathways are thought to be major causes of aging and related diseases [131].

### Nicotinamide adenine dinucleotide (NAD^+^), nicotinamide riboside (NR), and nicotinamide mononucleotide (NMN)

**Scheme 4:**
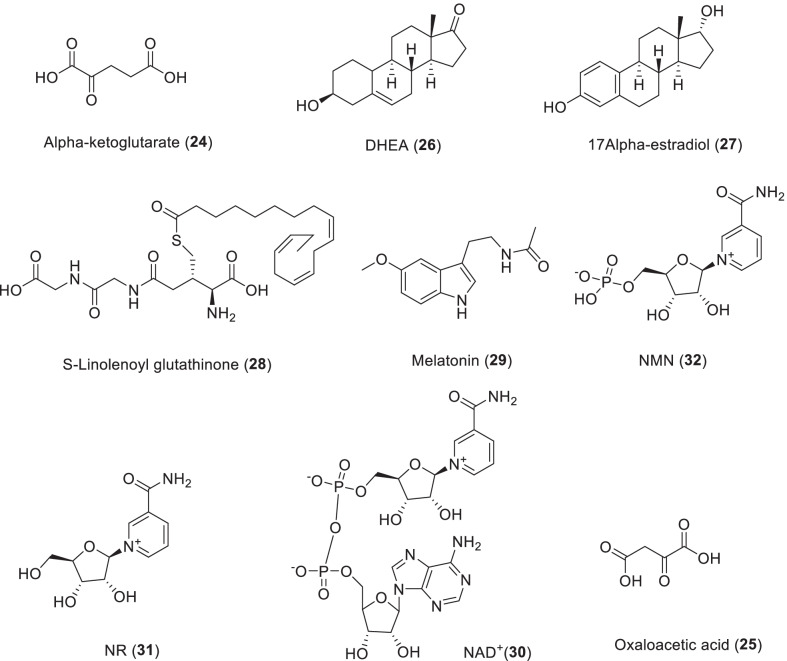
The structures of endogenous substances with the activity of life span extension

Nicotinamide adenine dinucleotide (NAD^+^, **30**) is an indispensable pyridine nucleotide that is used as a cofactor and substrate for many key cellular processes including DNA repair, epigenetic regulation of gene expression, oxidative phosphorylation, ATP production, intracellular calcium signal transduction, and immune function. It is well known that NAD^+^ concentration increases with reduced energy load. These activities include fasting, glucose deprivation, calorie restriction and exercise. However, with the exception of pellagra, NAD^+^ levels decreased in high-fat diet animals and during aging and cellular senescence [132]. Considering that NAD^+^ levels increase with increased life or healthspan and decrease with accelerated aging and/or reduced healthspan, it suggests that reduced NAD^+^ levels may be a major aspect in the aging process [133]. Therefore, NAD^+^ supplementation and its precursors may be a potential therapeutic strategy that mediates protection against inflammation and the accumulation of highly volatile reactive oxygen species (ROS) during aging. Oral supplementation of NAD^+^ and NADH did not show a significant increase in NAD^+^ levels in plasma or tissue, which may be due to poor bioavailability due to the inefficiency of gut NAD^+^ metabolism. At present, intravenous infusion of NAD^+^ is clinically recognized as the only effective means to improve the level of systemic NAD^+^. However, it is expected that some alternative NAD^+^ precursors, including NA, NAM, NMN, NR and NAR, may provide some benefits (Scheme 4) [134].

The discovery of Sirtuins, a deacetylase dependent on NAD and a deacetylase associated with longevity, has opened up a whole new field of aging research [135]. Interest in using the NAD/Sirtuin pathway to especially treat brain aging has greatly increased recently, and therapies based on this biological principle become clinically available sooner or later [136].

Recently, NR (**31**) has been identified as an NAD^+^ precursor vitamin with unique oral bioavailability in mice and humans [137]. Blood NAD^+^ levels have been shown to increase 2.7 times after 7 days of a single daily dose of NR (1000 mg). Another study showed that NMN (**32**) is metabolized extracellularly to produce NR, which is then converted into NAD^+^ intracellular [138]. In metabolically impaired mice, the addition of NR was related to increased SIRT1 expression, reduced oxidative stress level and enhanced mitochondrial function [139]. In a fly model of Parkinson’s disease, NR supplementation reduces the loss of dopaminergic neurons and improves motor skills [140]. NR supplementation reduces tau phosphorylation and enhances cognitive function in a mouse model of Alzheimer’s disease with DNA repair defects [141].

Another study showed that NMN (**32**) supplementation was sufficient to promote mitogenesis in nematode neurons and improve cognitive decline caused by Alzheimer’s disease [142, 143]. In a rat model of Alzheimer's disease, NMN reduced Aβ aggregation, enhanced spatial memory, and increased neuronal survival, in part by reducing ROS [144].

## Drugs with antiaging effects

### Acarbose

Acarbose (**33**) is an α-glycosidase inhibitor. It substantially increased the average and maximum lifespan in male mice at 1% concentration [145]. Harrison et al. reported that acarbose significantly extended the median lifespan of genetically heterogeneous mice (22% in males and 5% in females). Increased lifespan was related to increased short-chain fatty acid content in feces, suggesting the involvement of the gut microbiota [146]. In rats, acarbose lowered body weight and body fat and increased glucose metabolism without decreasing food intake. Similar to metformin, acarbose is accepted worldwide to treat diabetes and therefore has a high safety and efficacy profile. Thus, its effect on lifespan can be evaluated in healthy subjects. Acarbose is known to reduce blood sugar levels in the body. A post hoc analysis showed that weight loss with acarbose therapy was not based on glycemic control status, but relied on baseline body weight [147]. Acarbose significantly reduces body weight and abdominal obesity, decreases epicardial fat, intima-media thickness, C-reactive protein levels, and improves HDL (high-density lipoprotein) levels in patients with metabolic syndrome [148]. Acarbose decreased key markers (Iba1^+^ microglia, tumor necrosis factor-α, and glial fibrillary acidic protein) of hypothalamic inflammation, while long-term treatment of acarbose (from 3 to 9 months, 20 mg/kg/day) enhanced memory during aging in SAMP8 mice [149, 150].

One of the dominant views in gerontology is that impaired glucose regulation accelerates the aging process. This idea predicts that maintaining the integrity of glucose metabolism will delay aging. Acarbose is a potent inhibitor of small intestinal alpha-glucosidase, which reduces the breakdown of complex carbohydrates into glucose, thereby decreasing glucose uptake [6].

### Aspirin

Aspirin (**34**) is a drug used to relieve pain and inflammation. It was one of the drugs tested by the National Institute on Aging’s Intervention Trial Program [34, 151]. Female mice given aspirin lived especially longer. In the same report, nordidehydroguaiaretic acid as an antioxidative agent also showed a longevity effect, as mentioned earlier [34]. In *Caenorhabditis elegans*, aspirin also extends life span, prevents oxidative and heat stress, reduces intracellular protein aggregation, and prevents age-related locomotor decline [152, 153].

### (−)Deprenyl

(−)Deprenyl (**35**) is an inhibitor of monoamine oxidase B (MAO B). It is known to up-regulate the activity of antioxidant enzymes SOD and CAT in the dopaminergic region of the brain. The drug is also the only chemical that has been repeatedly found to extend the lifespan of rats, mice, hamsters and dogs in animal models. In addition, the drug can also enhance antioxidant enzyme activity in tissues outside the brain, such as the adrenal glands, heart, kidneys, and spleen. The results showed that deprenyl can activate a variety of humoral factors (interferon-γ, tumor necrosis factor-α, interleukin-1β, 2, 6, etc.) and enhance the function of natural killer cells. These new observations of (−)deprenyl up-regulating SOD and CAT activities in the brain and in extra-brain vital organs involve anti-tumorigenic and immunomodulatory effects, it may be better explained by reports of significantly longer lifespan in experimental animals in the past. These combined drug effects may cause homeostasis regulation of the body’s neuro-immune-endocrine axis for protection against aging [154].

### Metformin

The history of metformin for anti-diabetes (**36**) can date back to the seventeenth century, when the guanidine compounds were found in extracts from the leaves of French lilac. The anti-glycemic effects of French lilac *Galega officinalis* were first described in 1653 [155, 156]. Werner and Bell synthesized metformin and related biguanide compounds in 1922, paving the way for its widespread use in humans for type 2 diabetes as a first-line therapeutic agent [157]. In 1957, the drug was approved for the therapy of diabetes through the efforts of Jean Sterne, a French doctor. Observational studies have shown that diabetic patients treated with metformin have improved survival, even compared with non-diabetic controls [158]. Observational data in humans further reinforce metformin’s role in preventing age-related degeneration and cancer [159]. Metformin has been used as an anti-aging drug in model organisms and humans due to its proven safety record in more than 60 years of clinical use, its efficacy in protecting the heart, and its potential value in preventing and treating cancer [160, 161]. The lifespan-extending effects in *C. elegans* and mice for metformin have been reported [162–164]. A computational model study provides evidence that SIRT1, an NAD^+^ related histone deacetylase, may be the target of metformin [165]. Metformin may also affect the epigenome indirectly by regulating metabolite levels, which are known to change activity of histone and DNA-regulating enzymes. Metformin is known to impact cellular levels of NAD^+^, ATP, and tricarboxylic acid intermediates, as well as AMPK, which affect epigenome modification enzyme activity [166]. It was reported that metformin promotes the lifespan of nematodes by altering microbial metabolism of folate and methionine [167]. The Metformin in Longevity Study, a double-blind, placebo-controlled crossover clinical trial launched in 2014 with 14 human participants, the aim was to determine whether a daily dose of 1700 mg of metformin could improve the expression of the more youthful gene in older adults with impaired glucose tolerance. A larger double-blind, placebo-controlled, multicenter trial targeting aging with metformin plans to enroll 3000 patients between the ages of 65 and 79 as the primary endpoint before any aging-related diseases develop. The subjects will take metformin with a dose of 15000 mg daily for 6 years, with an average follow-up of more than 3.5 years [168].

Metformin has been used for diabetes with a good safety record for more than 60 years. There is growing evidence in humans and in preclinical models that it showed beneficial effects to reduce the risk of age-related diseases. These properties of metformin have drawn the attention of a large number of researchers including industry, to develop anti-aging therapies as an indication of metformin for humans. While on the surface this seems reasonable based on the safety and tolerability of the drug, there is still a lot we don’t know about metformin’s mode of action, especially when it comes to aging. Aging is a heterogeneous phenomenon. Different individuals in the same population respond differently to metformin. Therefore, there is a need for large-scale, multicenter, randomized, placebo-controlled trials to further elucidate the anti-aging effects of metformin. Recommendations to treat older adults with metformin may require a personalized, precise approach. Once we have better biomarkers of the beneficial effects of metformin on human health and longevity, we can develop these products [155].

### Minocycline

Minocycline (**37**) is an antibiotic with antioxidant, anti-inflammatory, and neuroprotective properties independent of its antibacterial activity, inhibiting the formation of kynurenine (KYN) in tryptophan (TRP). Since minocycline is the only FDA-approved drug with inhibitory effects on TRP-KYN metabolism, it is interesting to investigate the effects of minocycline on lifespan and healthspan in a fruit flies model. Minocycline (0.87 mM) extended the mean, median, and maximum lifespan of wild-type fruit flies (*Drosophila melanogaster*). Minocycline (0.87 mM) stimulates vertical climb of male flies. Minocycline dose-dependently reduces the number and survival rate of offspring pupae. Minocycline may be a promising candidate for anti-aging intervention and treatment of aging-related medical and psychiatric disorders [169]. It treated flies lived significantly longer (101 ± 1.33 days) than ascorbic acid treated flies and control flies (42.3% and 38.4%, respectively). From the 3rd week to the 9th week of treatment, the exercise activity of minocycline treated flies was also significantly higher than that of control flies and ascorbic acid treated flies [170]. Minocycline supplementation significantly extended the lifespan of S and W^1118^ fruit fly strains. The extension effects of lifespan for drug were not related to decreased dietary intake or reduced fertility in female, but to increased resistance to oxidative stress (hydrogen peroxide). Of note, the effects of minocycline on longevity and antioxidant stress were largely eliminated in FOXO null mutants, and pharmacological treatment improved FOXO activity [171].

### Statins (Simvastatin)

The cholesterol-lowering drugs, statins, can affect lifespan by reducing the risk of ischemic heart disease. Treatment of *Drosophila* with simvastatin reduces arrhythmias associated with aging and extends lifespan [172]. Cholesterol-lowering drugs such as simvastatin (**38** or other statins) may act directly on antioxidant pathways or telomere shortening to regulate health during aging [173, 174]. However, simvastatin has not been reported to promote longevity in mice [151]. Despite the discouraging results, many case–control studies and clinical trials suggest that statins have shown considerable potential for statins in health and prevention of age-related diseases (e.g., dementia, cancer, etc.) [173].

Simvastatin (10 or 20 mg/kg for 4 weeks) reduced Aβ plaques, neurotoxicity, and proinflammatory cytokines and improved memory in the Morris water maze and Y-maze in diabetic mice [175]. In a mouse model of Alzheimer’s disease, it reduced Aβ neurotoxicity and upregulated brain-derived neurotrophic factor levels in the hippocampus and dentate gyrus [176]. Simvastatin was reported to reduce neuroinflammation and Aβ plaques, improve cerebral blood flow, and alleviate oxidative stress in amyloid precursor protein mice [177]. Several large-scale studies for the treatment of dementia and meta-analyses have shown the potential of statins, but conflicting results have also been reported [178]. A postmortem study showed that statin users had a reduced risk of developing the classic pathology of AD (nerve protuberant plaques or tangles) [179]. Patients between 78 and 85 years of age who took statins lived 2 years longer [180]. Cholesterol levels were not associated with increased longevity in older adults [181].

### Others (Celecoxib, Doxycycline, Enalapril, Metoprolol, Nebivolol)

**Scheme 5:**
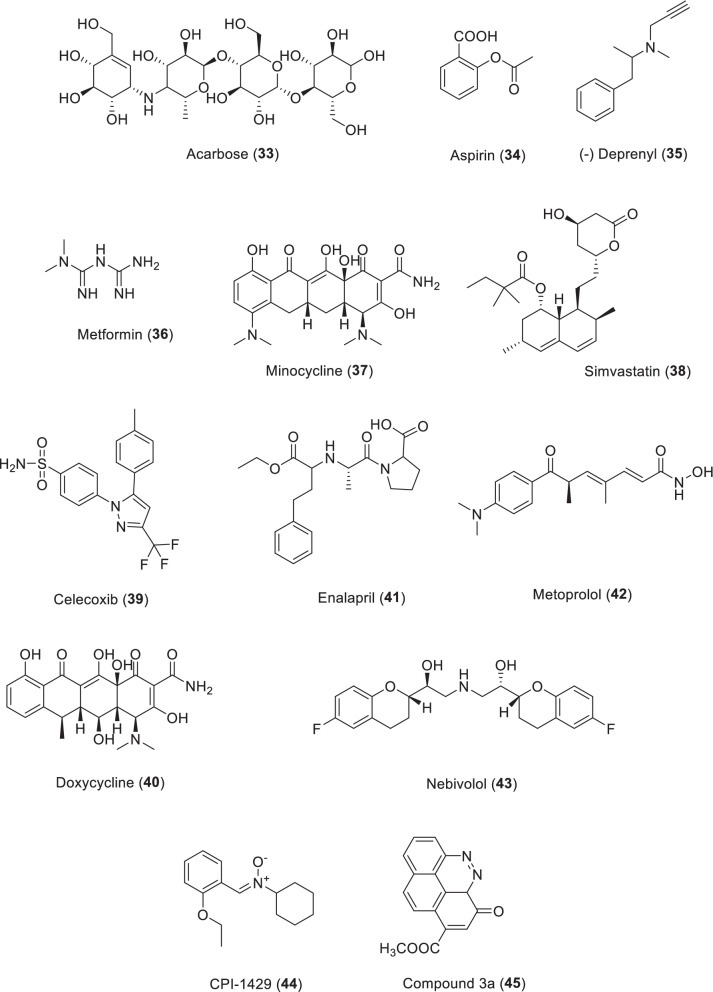
The structures of drugs and synthetic compounds with antiaging effects

Celecoxib (**39**) is a non-steroidal anti-inflammatory drug originally developed as a cyclooxygenase-2 inhibitor. It was found to prolong the lifespan of the worms in a dose-dependent manner. Doxycycline (**40**) is a broad-spectrum antibiotic of tetracycline type, which can prolong the life span of nematodes and *Drosophila melanogaster* [182–184]. In healthy (normal blood pressure) rats, long-term administration of enalapril (**41**, an angiotensin converting enzyme inhibitor) was associated with significant weight loss and a longer life span [185]. Metoprolol (**42**) and Nebivolol (**43**) as beta-blockers increased the mean and median life span of male mice by 10% and 6.4%, respectively, and extended the life span of Drosophila (Scheme 5) [186].

## Synthetic compounds with antiaging activity

### Nitrons (4-hydroxy phenyl *N*-tert-butylnitrone, CPI-1429)

Nitrone-based free radical traps have important potential in treating neurodegenerative diseases and prolonging life span. It first attracted scientific attention that the action of free radical trapping of these compounds is the property. A new nitrone, CPI-1429 (**44**), has been reported to show an ability to prolong life span even when the compound is introduced in older animals [187]. The possible effect of CPI-1429 on learning and memory impairment in C57BL/6 mice during normal aging was tested. Because the use of the learning/memory paradigm requires extensive training and testing, the elderly mice in these experiments (23–24 months) were maintained for up to 27 weeks with long-term treatment of the compound. When survival analyses were performed on the treated and control groups, it was clear that the mortality of mice treated with CPI-1429 was significantly reduced over a relatively wide dose range. The treatment had no effect on the mice’s weight, but was associated with improved performance on learning and memory tasks [188, 189].

### Pyridoperimidine derivatives (compound 3a)

Compound 3A (**45**) extended the longevity of wild-type nematodes under standard laboratory conditions. The heat resistance and chemical stress resistance of wild *C. elegans* were significantly improved by its treatment. In addition, the treatment with **45** worms significantly reduced the formation of advanced glycation end products in a reverse dose-dependent manner [190].

The antiaging effect of MHY2233 was reported recently [191]. It is mainly due to SIRT1 deacetylase activity and upregulation of SIRT1 expression. MHY2233 was found to be highly effective in reducing replicative senescence and oxidative stress-induced senescence in EPCs. Moreover, MHY2233 increases the functional activities of senescent EPCs, such as cell proliferation, migration, and angiogenesis. The novel compound MHY2233 can be used as a potent therapeutic target for the treatment of aging and age-associated cardiovascular diseases. A series of 3,5-disubstituted isoxazoles were synthesized. The biological evaluation of the deprotected compounds showed a correlation of their antioxidant properties with stress resistance in human primary fibroblasts (in vitro model) and with the extended longevity of the nematode *C. elegans* (in vivo model) [192].

The discovery of anti-aging agents from synthetic compounds is still relatively little reported. The main reason for this is that anti-aging targets are not yet widely accepted from a drug discovery perspective. In addition, there is uncertainty at this stage regarding the safety of synthetic compounds for life-extending drugs. However, in the long term, if more and more agents from existing clinically safe drugs are used and proven in clinical applications for health span extension, it is likely that more anti-aging agents from synthetic compounds will emerge in the future.

## Intervention and model systems for the evaluation of antiaging compounds

### Animal test (nematodes, fruit flies, mice)

Much of our understanding of the molecular pathways that regulate the life span comes from studying *Caenorhabditis elegans*. The discovery of *age-1*, the first gene in nematodes to regulate life span, led to an explosion in the study of aging in this system [193, 194]. The finding is important because it shows that life span, like other developmental processes, is controlled by genes. Until the discovery of a second age mutant, *daf-2*, the study of aging in *C. elegans* seemed to become more popular and of more interest to the public [195]. Recently, hundreds of genes that regulate the lifespan of *C. elegans* have been identified. Analysis of these genes revealed that in addition to the IIS pathway, signaling pathways also regulate life span, including sirtuins, the target of rapamycin (TOR), Jun kinases (JNK), protein translation, and mitochondrial signal transduction [196].

Because of their short lifespan and ease of culture, *C. elegans* is rapidly becoming a model for testing the activity of compounds in aging and age-related phenotypes. Indeed, not only can nematodes be used to test the biological effects of many compounds, the large number of genetic tools available in *C. elegans* makes it a powerful system for studying the mechanisms of pharmacotherapeutic action [197]. Due to its relative simplicity, rich biological knowledge, and the large number of genetic tools available, it turns out to be a very attractive organism in the field of biopharmaceutical research. This nematode has proved to be very useful in determining the biological activity of compounds and in determining their mechanism of action. Moreover, its rapid growth and high fecundity make it very suitable for high-throughput chemical screening. As a pharmacological tool, it has recently been used to study small molecules that affect development, such as inhibition of neurotransmission and promotion of neural regeneration. It is important to emphasize that this nematode has a particularly important feature in that it feeds on live cultured bacteria. This means that there is an inherent possibility in observing its response to a certain chemical due to the fact that it interacts with bacteria in some way. This possibility should always be looked at. This can be achieved by growing and maintaining nematodes on killed bacteria or by using sterile culture conditions in chemical reaction tests [198].

**Fig. 1:**
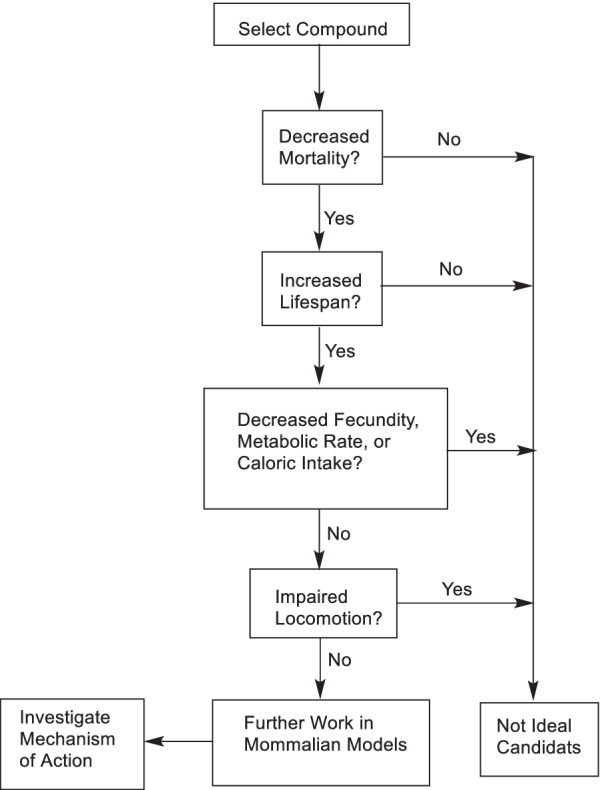
Proposed algorithm to screen antiaging agents

A good model system for human-related aging research would include genes that are highly conserved with humans, capable of being genetically manipulated, multiple life stages, and sufficiently short lifespans and generation times to allow effective monitoring within a reasonable period of time. *Drosophila melanogaster* meets these criteria, and it provides a suitable model system for screening anti-aging compounds. Humans and *Drosophila melanogaster* share many conserved physiological and biological pathways. In addition, because *Drosophila melanogaster* has a long history of use as a model system in many areas of research, there are a variety of genetic strains with different lifespans to choose from. Not only is it important to validate compound activity in flies with multiple genetic backgrounds, but genetic manipulation of *D. melanogaster* also allows for the development of accelerated assays based on age-dependent expression of molecular biomarkers in combination with lethal toxins. In addition, the availability of many transgenic models (e.g., fruit flies lacking or having a particular gene function) allows mechanistic studies to assess whether the action of a specific compound depends on a particular pathway. Finally, the use of short-lived organism models, such as *D. melanogaster*, is a key in anti-aging research projects, as lifespan analysis represents a speed limit for experimental steps. The causes of aging are a complex issue because many factors influence aging, including genetics, environment, metabolism, and reproduction. These multiple factors make it particularly difficult to evaluate anti-aging compounds, which requires a good model system to evaluate those potential anti-aging agents. The model systems used should be able to reflect the complexity of human aging so that the results can be extrapolated to human studies. However, they should also provide the opportunity to minimize variables in order to accurately interpret experimental results. In addition to the positive effects on longevity, the effects of the compound on physiological confounders of aging in model organisms need to be assessed, including fecundity and healthspan (the lifespan of an organism that is generally healthy and free of serious or chronic disease). **Fertility** is considered to be the main confounder of aging in *D. melanogaster.* Female flies exposed to toxic substances are known to reduce their dietary intake and reproduction and to artificially extend their lifespan. Therefore, drugs that reduce fecundity by reducing food intake and thus gain longevity may not be effective candidates for the eventual treatment of human aging and should be excluded during the screening process. **Metabolic rate** is another important potential physiological confounder that must also be looked at. For example, lamotrigine was found to reduce mortality and extend lifespan in both male and female flies, but it reduced metabolic rate and impaired physical performance through locomotor activity (Fig. 1) [199].

With the ultimate focus on promoting human health, it is critical to test genetic interventions in mammals, and mice have proven to be the best mammals for this task. Lifespan studies in mice are resource-intensive and can take up to 4 years to complete. Therefore, it is important to follow a set of science-based criteria to ensure that reliable study results are obtained that are based on statistically significant data so that other laboratories can replicate them. To successfully complete a mouse lifespan study, a number of requirements must be met. (1) The study subjects should be mice of uniform genetic background. Many transgenic and knockout strains are generated in mixed backgrounds. Generally, when strains are generated, the progeny of the breeding line will return to the inbred line, but this requires at least 10 generations of backcrossing to become homologous. The reality is that time and resources are limited, and after only a few generations of backcrossing, mice are tested in mixed backgrounds. This increases the genetic variability of each mouse and reduces statistical significance. Researchers should be encouraged to use long-lived inbred or hybrid lines whenever possible, but if a particular genetic mutation happens to occur in an essentially short-lived background line, exploratory studies are still feasible even if it is not an inbred line. If the initial study is promising, additional follow-up supplemental studies can be conducted by returning to a longer-lived line. (2) Survival must be measured in a statistically significant large sample of mice. The use of large numbers of animals has two important implications for studying longevity. First, larger sample sizes reduce the effect of each animal on population survival; that is, results are less likely to be skewed by any isolated observations, so that transgenic survival curves are more reproducible and accurate. Second, the large sample size allows us to analyze the survival curves of the animal models in more detail. (3) Appropriate breeding programs free from pathogen contamination must be provided. Management of aging populations is critical and, if done incorrectly, can make survival data unreliable, especially when assessing the effects of modified aging. (4) Males and females should be listed separately. Studies of mouse lifespan must take into account gender differences. (5) The end of life must be defined. Lifespan should be determined by allowing mice to live as long as possible. Mice should be killed only if it is determined that they would die within a few hours without intervention. The most reliable indication is the lack of response to being touched. Continuous and frequent observation of the animals 7 days a week is essential in this regard. End-of-life criteria must meet the guidelines of the animal care facility and ensure that no significant number of autolytic mice are found dead in the cage, otherwise an adequate pathological assessment will not be possible. (6) Physiological and pathological assessments can be included to expand the data. While lifespan data are a critical first step in determining whether modifications retard aging, pathological and physiological information can help determine the effect of modifications on aging [200].

### Autophagy induction

Autophagy is an evolutionarily conserved cytoplasmic degradation system in which various substances are sequestered by double-membrane structured autophagosomes and sent to lysosomes for degradation. Because of its diverse targets, autophagic activity is critical for the stability of the intracellular environment. New genetic evidence suggests that autophagy plays an important role in regulating animal lifespan [201]. Genes associated with autophagy have been shown to be critical for lifespan in a variety of organisms including yeast, flies, nematodes, and mice. There is also compelling evidence that induction of autophagy leads to increased lifespan, while inhibition of autophagy has the opposite effect [202–205]. Polyamines, particularly spermidine, are among the most potent inducers of autophagy. Interestingly, activation of autophagy is generally associated with the inhibition of IGF and mTOR signaling pathways, and inhibitors of these pathways such as rapamycin are widely used as inducers of autophagy. Activation of autophagy is also triggered during periods of caloric restriction. The widely popular resveratrol is a “healthy” supplement whose aging-inhibiting properties are thought to be mediated by activation of sirtuins (NAD^+^-dependent protein deacetylase family) SIRT-1, which is also involved in the induction of autophagy [206]. In the last decades, evolutionarily conserved molecular mechanisms have been identified in several model organisms, including yeast, worms, fruit flies, and mice, that delay aging and extend lifespan in animals. These pathways include, for example, inhibition of the insulin/IGF-1 signaling pathway, dietary restriction, inhibition of the TOR signaling pathway, removal of germ cells, and attenuation of mitochondrial respiration. Extensive studies of the downstream mechanisms of each longevity pathway have shown that many different factors or biological processes act as regulators in each pathway, although some act together. Of particular note, recent studies from nematodes suggest that autophagy is one of the converging downstream mechanisms of all these longevity patterns [201].

**Fig. 2:**
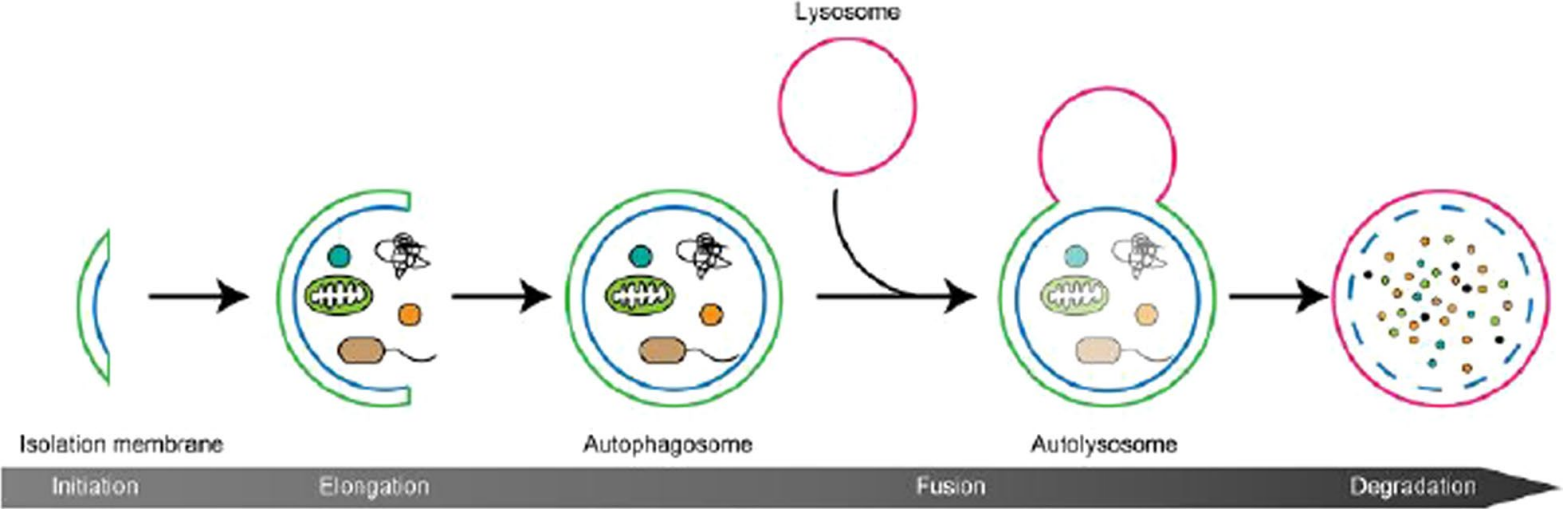
Overview of macroautophagy [201]

Autophagic mechanisms can be subdivided into three categories: chaperone-mediated autophagy, micro-autophagy and macro-autophagy. Chaperone-mediated autophagy is a partner-dependent selection that detects specific consistent protein sequences through chaperone complexes and transfers them to lysosomes. Micro-autophagy is the direct invasion of lipids, proteins or organelles by lysosomal membranes mediated by acid hydrolases. Macro-autophagy plays a key role in intracellular quality control by removing dysfunctional organelles and thus protecting the whole cell from death (Fig. 2) [207]. There is no doubt that autophagy has powerful anti-aging properties, but mechanistically, how this positive effect is achieved is still unknown. It is hypothesized that three main functions of autophagy may contribute to cellular protection: (a) buffering cellular stress in the presence of fluctuating nutrient supply by strengthening substrates that provide bioenergetic and anabolic responses; (b) removal of dysfunctional and harmful organelles, including uncoupled mitochondria; and (c) removal of easily aggregated and potentially toxic proteins. At the organismal level, these functions can be extended by immune regulation of autophagy, cellular autonomy and immune surveillance-mediated suppression of tumorigenesis [208].

### Calorie-restriction

In 1935, McCay et al. published the seminal paper showing that restricting food intake in rats to levels well below that of ad libitum feeding significantly extended life span [209]. Since then, it has been found that reducing food intake from 30 to 50% from ad libitum can extend the life span of many different species of rats and mice [210]. In addition, food restriction has been found to extend lifespan in non-mammalian species, including yeast, nematodes, and fruit flies as model animals [211–213]. Although there was no conclusive evidence at the time that food restriction could extend lifespan in primates, there was evidence that it could reduce risk factors for aging-related diseases in rhesus monkeys and humans [214, 215]. Subsequently, caloric restriction (CR) was shown to extend the lifespan of rhesus monkeys, which belong to the same primate group as humans [216]. Yet another report showed that CR improved health but did not extend lifespan [217]. Because these studies did not use the same dietary conditions, it is difficult to directly compare these results [218]. Furthermore, in primates, CR is thought to extend lifespan, depending on dietary conditions. Although this series of reports represents a landmark achievement, it is difficult to implement CR in the same way. In recent years, research has progressed in finding and evaluating compounds with similar effects to CR administered orally [219]. There are studies demonstrating the feasibility of CR in humans (for at least 2 years) and its beneficial effects on longevity risk factors and cardiometabolic risk factors [220].

**Scheme 6:**
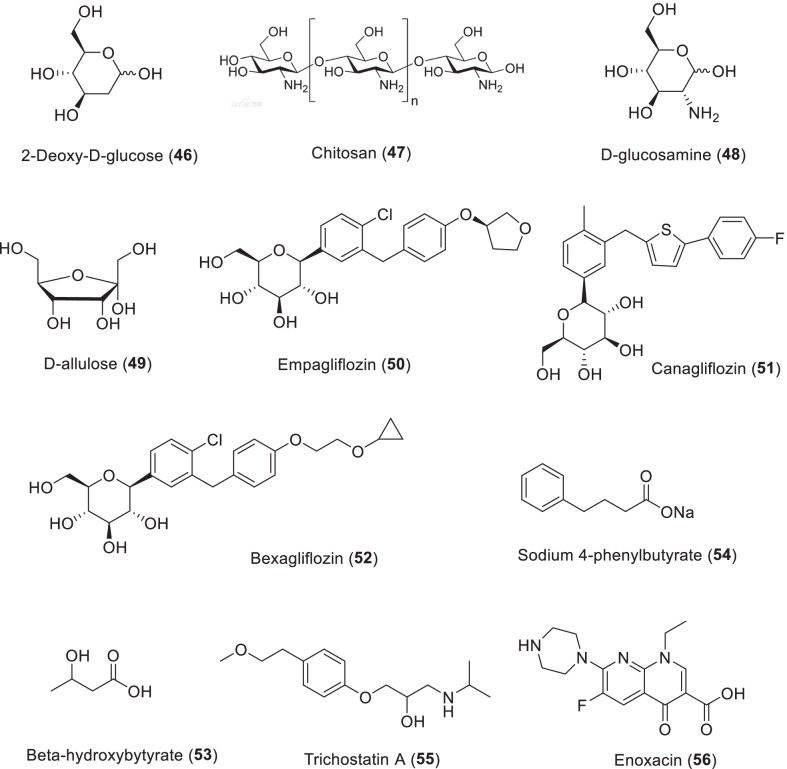
The structures of caloric restriction mimics

Although CRs can extend human lifespan, they are also difficult to implement in humans in the long term. Therefore, there is a need to develop a method or compound that mimics or reproduces the effects of CR without limiting the amount of food. The concept of CRMs was proposed by Lane et al. in a study of 2-deoxy-d-glucose (2DG, **46**) and was demonstrated in rat experiments. CRMs exhibit systemic effects of CR, including a wide range of compounds, bariatric surgery, and exercise [221]. Downstream and upstream CRMs have been identified. Downstream CRMs act on intracellular signaling systems and play the same role as CRs in downstream pathways: metformin (**36,** AMPK activation), rapamycin (**5**, mTOR inhibition), resveratrol (**6**, Sirtuin activation), polyamines (epigenetic control), oxaloacetate (**25**, REDOX equilibrium). Upstream CRM, on the other hand, uses a mechanism of action that targets the energy metabolic system and sends signals upstream to mimic CR: chitosan (**47**, glucose reduction), acarbose (**33**, glycosidase inhibition), 2-deoxy-d-glucose (**46**, glycolytic inhibition), d-glucosamine (**48**, glycolytic regulation), d-allulose (**49**, glycolytic improvement), SGLT2 inhibitor (empagliflozin **50**, canagliflozin **51**, bexagliflozin **52**) (Scheme 6). All upstream types of CRMs are thought to affect glucose utilization [222].

2-Deoxy-d-glucose (**46**, 2DG) is a glucose derivative in which the 2-position hydroxyl group of glucose is replaced by a hydrogen atom. 2DG is not metabolized by glycolysis and was the first proposed dietary restriction mimetic [221]. It is recognized to delay age-related dysfunction and prolong lifespan by inhibiting glycolytic activity.

d-Glucosamine (**48**, GlcN) is the building block of chitosan and chitin and is produced in nature by arthropods, fungi, and cephalopods. GlcN is produced industrially by hydrolyzing the exoskeleton of crustaceans, which is mostly composed of chitin. GlcN is a popular dietary supplement for the prevention and treatment of osteoarthritis in humans. Weimer et al. reported longevity effects of GlcN in nematodes and mice. The authors concluded that these effects were caused by impaired glucose metabolism [223]. GlcN enters cells via glucose transporters, inhibits glycolysis, and induces stored fat metabolism and mitochondrial respiration via AMPK. Increased respiration can lead to the temporary formation of ROS, resulting in increased antioxidant enzyme activity, resistance to oxidative stress, and survival. Oral administration of GlcN has been reported to affect carbohydrate metabolism and reduce body fat in rodents, contributing to increased resistance to oxidative stress and subsequent activation of AMPK. The compound has also been reported to induce autophagy in mammalian cells through a signaling pathway independent of mTOR [222]. In a clinical trial, oral administration of GlcN was used to improve vascular endothelial function by modulating intracellular redox status [224]. According to a large epidemiological study of consumers of various dietary supplements, the use of GlcN was associated with a reduction in overall mortality [225].

d-Allose (**49**, d-Alu) is the C-3 epimer of d-fructose, a rare hexose found in limited quantities in nature. However, this compound is marketed as a zero-calorie functional sweetener and is easily produced in large quantities from d-fructose. Numerous studies have shown that d-Alu has various effects such as anti-hyperglycemic and anti-obesity. d-Alu can prolong the life span of nematodes [226]. Similar to GlcN and 2DG, d-Alu enters cells through glucose transporters, inhibits glycolysis, and induces metabolism of stored fats and mitochondrial respiration via AMPK. Increased respiration leads to a transient upregulation of ROS production, resulting in increased antioxidant activity, resistance to oxidative stress, and viability [227]. Both d-Alu and GlcN contain a functional hexosaccharide with high safety and health benefits that are thought to extend lifespan.

### Epigenetic regulation of gene activity (DNA methylation, histone modification, miRNA regulation)

Scientists have worked tirelessly to elucidate the causal mechanisms underlying the phenotypic changes associated with aging. As the expression of many genes appears altered during aging, researchers have focused on the long-term effects of environmental stress on the regulation of gene expression. Importantly, epigenetic modifications are thought to play a key role in the aging process. Epigenetic changes are genetic variations triggered by the environment and include alterations in DNA methylation, histone modifications, noncoding RNAs, ribosomal localization, and transcription factor binding [228, 229]. DNA methylation is a biological process in which methyl groups are added to DNA molecules. Histone modifications include arginine methylation, lysine acetylation, lysine methylation, and serine phosphorylation. These modifications alter the extent to which DNA wraps around histones and the extent to which genes in DNA can be activated. Recently, several studies have highlighted the unique role of DNA methylation in aging [230, 231]. DNA methylation involves two distinct processes: in DNA, the addition and removal of the 5th methyl group of cytosine (5mC) or the 6th methyl group of adenine (6 mA) [232]. Studies have shown that DNA methylation can be used as an accurate biomarker to estimate “biological age” and thus to predict changes associated with aging. In recent years, great progress has been made in several potential methods for estimating biological age, with DNA methylation levels being the most promising [233]. Studies clearly show that genome-wide DNA methylation levels are associated with chronological age throughout the human lifespan. Some age-related DNA methylation changes occur in specific regions of the genome and are directional, suggesting the existence of differentially methylated regions associated with age. Thus, biomarkers based on DNA methylation can accurately estimate age, as has been demonstrated by many investigations involving tissues, individuals, and populations [234]. The reversibility of DNA methylation is the most interesting feature of epigenetic clocks, which suggests that they can be used to measure the effectiveness of anti-aging interventions [235, 236].

The link between lifespan and histone methylation has been revealed in a variety of experimental models, including yeast, nematodes, fruit flies, mice and humans [237, 238]. To date, the anti-aging and lifespan prolonging effects of HDAC inhibitors have only been investigated in one study of nematodes. The effects of endogenous ketone D-βHB (**53**, D-βHB) on senescence of nematodes were determined. Addition of D-βHB can prolong the mean lifespan of nematodes by about 20% [239]. In *D. melanogaster*, sodium 4-phenylbutyrate (**54**, PBA) demonstrated its life-extending potential [240]. Feeding PBA significantly increased mean and maximum lifespan by up to 30–50%, while motor activity and resistance to stress were not diminished. The mean and maximum life span of fruit flies were significantly prolonged by single treatment of sodium butyrate (SB) (25.8% and 11.5%, respectively) [241]. Subsequently, other authors confirmed the life-extending ability of *D. Melanogaster* treated by SB [242–244]. Trichostatin A (**55**, TSA) is another HDAC inhibitor with a wide range of epigenetic activity, which was widely used. The phenotypic and epigenetic effects of TSA treatment were very similar to those of SB treatment of *D. melanogaster*. Increases in mean and maximum lifespan were observed as a result of one-off and continuous TSA treatment [241, 245]. In some rodent models, HDAC inhibitors treatment has produced significant anti-aging and healthy lifespan effects, but direct effects on longevity have not been demonstrated. HDAC inhibitors are thought to have clinical potential in treating and/or preventing a number of chronic pathological diseases, including cardiovascular disease, cancer, metabolic and neurodegenerative diseases such as AD, PD, Huntington’s disease, inflammation, immune response invasion, and arthritis. To avoid adverse consequences, the development of stage-specific, tissue-specific, and HDAC specific inhibitors is particularly promising, and considerable research work is currently underway to find such compounds [246].

MicroRNAs (miRNAs) are short non-coding RNAs that are involved in post-transcriptional regulation of protein-coding genes. miRNAs regulate lifespan and aging in a variety of organisms. It has been confirmed that Mir-83 regulates the life span of *C. elegans*. miR-83 mutants showed life-prolonging activity, and the overexpression of miR-83 was adequate to reduce the extended lifespan of the mutants [247]. A highly conserved miRNA miR-124 was significantly up-regulated in *Caenorhabditis elegans* treated with *Astragalus* polysaccharides (APS). Overexpression of miR-124 induced the extension of nematode lifespan, and vice versa, suggesting that miR-124 regulates nematode lifespan [248]. miR-184, miR-125 miR-100 and let-7 were found to be candidate miRNAs involved in the regulation of aging by comparing the miRNA expression of the long-lived fruit flies population fed a low nutrition diet with that of the normal control group fed a high-nutrient diet. It was found that the prevalent, adult-specific overexpression of miRNAs in these individuals resulted in obvious changes in lifespan and/or fat metabolism. Most impressively, adult-specific overexpression of let-7 in female neural tissue increased the average lifespan of fruit lies by approximately 22% [249]. The abundance of miRNAs in the miR-58 family is up-regulated in long-lived daf-2 mutants, suggesting that these miRNAs are effectors of insulin signaling in nematodes. miR-58 is regulated by the insulin signaling pathway and is partially required for life extension mediated by decreased insulin signaling, dietary restriction, germinal cell ablation, and mild mitochondrial dysfunction. Daf-21, isw-1 and ins-1 mRNA were further identified as endogenous targets for miR-58 [250]. Enoxacin (**56**) has been reported to extend lifespan by lowering miR-34-5p levels, interfering with redox equilibrium and promoting healthy lifespan [251]. The role of endogenous siRNA in aging has not been studied in comparison with miRNA. By combining nematode sequencing with genomic and genetic approaches, the unprecedented role of endogenous siRNA molecules in protein state maintenance and lifespan extension in reproductive animals is revealed [252].

### GH/IGF-1 axis inhibition

The studies performed in animal models have demonstrated that reduced IGF-1 levels or IGF-1 activity can prolong lifespan. In addition, gene polymorphisms of human IGF-1 receptor have been found to relate to exceptional longevity [253]. A study by Barzilai et al. displayed that low IGF-1 concentrations in plasma anticipated survival in a population of long-lived individuals (especially women with a history of cancer) [254]. In dwarf, long-lived mice short of the growth hormone receptor had low IGF-1 levels, were insulin-sensitive, and had a reduced risk of cancer and diabetes despite their obesity [255–257]. Importantly, similar results have been described in patients with growth hormone receptor-deficient Laron syndrome (LS). On this subject, no formal aging research has been conducted in LS patients; however, they are kept from diabetes mellitus and fatal tumors [258, 259]. Thus, pharmacological interventions that directly reduce IGF1 levels in adults may improve health and prolong lifespan.

### Inflammation inhibition

Chronic, low-level inflammation is thought to be a main feature of aging. Many age-related diseases, genes and pathways that modulate inflammation are candidate targets for fighting inflammation [260]. Despite its importance, the details of the mechanism that triggers the most important stimulus of inflammation remain unknown. Circulating mitochondrial DNA (mtDNA), known as foreign nucleic acid by immune sensors, is a strong inflammatory stimulus that increases with age [261]. Proinflammatory galactosylated *N*-glycan is one of the most dominebt biomarkers of human biological age [262].

### Transcripts from noncoding repetitive elements (Res)

Advances in transcriptomics, such as RNA-seq, are yielding important new insights into the many genes and pathways involved in the “aging signature” and the broader health output [263]. Most studies have concentrated on the coding sequences of small portions of the genome. Despite growing evidence that noncoding, repetitive fragments (Res, 60% or more of the genome) serve many important biological functions, they have long been neglected as “junk DNA.” Res include DNA transposons, retrotransposons, satellites, tandem and terminal repeats. The major parts of res are transposons (Tes), which include DNA transposons and retrotransposons, which have the ability to multiply, proliferate, and change genomic positions [264]. Most Res are located in chromatin and inhibited (inactive) regions of the genome, but recent reports suggest that some Tes become active during aging, possibly due to reduced chromatin structure/stability (e.g., histone dysregulation) [265]. Activation of these specific Tes may lead to genomic and cellular damage, stress, and inflammation leading to senescence [266]. Analysis of multiple RNA-seq datasets generated from human samples and nematodes revealed that most RE transcripts (a) increase progressively with age; (b) can be served to accurately predict age; (c) may be a good indicator of biological age. The strong RE/aging association observed is consistent with the increasing evidence that RE transcripts directly relate to aging and disease [267].

### Sestrin 2

Sestrin 2 is a stress response protein that is primarily regulated by p53 and acts as a cytoprotective agent against genotoxicity and oxidative stress. More importantly, Sestrin 2 is now characterized as a key regulator of cellular metabolism and an effective contributor to cellular homeostasis in disease and normal physiological states. Its powerful antioxidant effects have been found to be neuroprotective in neurodegenerative diseases closely related to oxidative stress, such as Alzheimer’s and Parkinson’s diseases. As inhibitors of mTORC1 and positive regulators of AMPK, sestrins play a protective role in various metabolic diseases such as atherosclerosis, cardiac hypertrophy, cancer, diabetes and obesity. Therefore, sestrins show enormous potential as a therapeutic target and a good prognostic marker for various diseases. To upregulate sestrins for therapeutic strategy design, it is important to decode the upstream and downstream pathways of sestrins, including antioxidant effects, elimination of hypoxic signaling and ER stress, mTORC1 inhibition/AMPK activation, autophagy activation, pro-survival effects on normal cells and suppress proliferation effects on cancer cells. Future research using transgenic animal models will include: conditional, organ-specific knockdown of Sestrin 2 and attempts to link Sestrin 2 levels (inpatient biopsy samples) to disease progression will help us to identify biochemical pathways regulated by Sestrin 2 in specific diseases. In addition, the screening and development of small molecule mimics or activators of Sestrin 2 using in vitro and in vivo will help to determine the therapeutic potential of Sestrin 2 as a drug target for various diseases [268].

### Senolytics

Cellular senescence occurs in many types of somatic cells and is a paradigm of counteracting polymorphism. It is a heterogeneous process caused by genetic, epigenetic and environmental factors [269]. It can be effective at inhibiting damage and carcinogenic signals, thereby improving the reproductive success of young organisms, but it becomes harmful with age, causing tissue response, function and regeneration to deteriorate with age. Cellular senescence is considered one of the markers of aging. It is believed to contribute to age-related dysfunction, aging and chronic diseases, including AD, atherosclerosis, cancer, chronic obstructive pulmonary disease, diabetes, hepatic steatosis, idiopathic pulmonary fibrosis, myocardial infarction, osteoarthritis, and osteoporosis [263, 270, 271].

Interfering with the pro-aging effects of cell senescence by completely eliminating senescent cells (SCs) or turning off senescence secretion mechanisms is now being recognized as a potential strategy for treating diseases of aging. Recently, several transgenic mouse models have been developed that make it possible to visualize, evaluate and destroy senescent cells in vivo. In these models, the promoter of the gene p16INK4A, a protein involved in cell cycle regulation, was used to control cell cycle transcription, inducing the death of senescent cells by using small molecules. Two similar but not identical transgenic mouse models have been used to selectively eliminate SCs by induction of an “activator” molecule: INK-ATTAC (by targeting activation of Ink-linked apoptosis in caspase mice) and p16-3MR mice. In p16-3MR mouse models, P16INK4A-positive SCs express a viral thymidine kinase that phosphorylates the ganciclovir prodrug, thereby transforming the compound into a toxic metabolite that causes mitochondrial DNA damage and cell death. The p16INK4a promoter can regulate the expression of fluorescent proteins in addition to the death-inducing products of transgenes. Thus, it is possible to visualize senescent cells in living animals [272].

Senolytic drugs, used to selectively induce apoptosis in senescent cells, have displayed promise in improving cardiometabolic health indicators and reducing aging-related diseases [273]. A landmark study showed that an “antiaging cocktail” consisting of quercetin and dasatinib substantially increased the survival and improved the physiological function of young and naturally aging mice that had been transplanted with cells. The results of this work suggest that administration of senolytic compounds can improve health and extend lifespan even in old age [274]. A recent landmark research showed that with the clearance of senescent cells, senolytic therapy was adequate to eliminate Aβ plaques, decrease neuroinflammation, and improve memory in AD model mice [275]. Thus, senolytic therapy may offer an exciting and promising approach for the treatment of neurodegenerative diseases and dementia.

Cellular senescence cannot be considered as a negative outcome alone. It also benefits various physiological functions such as embryonic development, insulin secretion from pancreatic β-cells, tissue regeneration, tumor suppression, wound healing, during aging [276–278]. Thus, cellular senescence may be a double-edged sword with advantages and disadvantages, and a similar warnings should be given when it is used as a therapeutic target [279].

### Sirtuins regulation

Sirtuins are a type of histone deacetylases whose enzymatic activity is dependent on NAD^+^ as a cofactor. Sirtuins have been reported to regulate various activities by controlling biogenesis, gene expression, metabolism, DNA repair, oxidative stress response, and mitochondrial function. Dysregulation of their action and/or expression may cause tissue-specific degenerative events involved in the development of multiple human pathologies, including cardiovascular disease, cancer, and neurodegeneration. In mammals, there are seven sirtuins that show different subcellular localizations. SIRT1, SIRT6 and SIRT7 are nuclear; SIRT2 is predominantly cytoplasm; SIRT3, SIRT4 and SIRT5 are mitochondria that go to the nucleus [280, 281].

Sirtuins are involved in the aging process in yeast, nematodes, flies and mice. In nematodes and flies, overexpression of sirt2 prolongs lifespan [282, 283]. In mice, overexpression of sirt6 in whole body prolonged the longevity of male mice by 15% [284]. Overexpression of sirt1 in the brain also prolonged the lifespan of mice [285]. In addition, activation of sirt1 reduces the risk of various aging-related diseases, including diabetes, cardiovascular disease, inflammation, and neurodegeneration in model animals [286]. In contrast, sirt6-deficient mice exhibit premature aging related to genomic instability and defective DNA repair at the cellular level [287]. Thus, activation of sirtuin activity is a target for lifespan extension and aging retardation.

Sirtuins have multiple functions in tissues. In white adipose tissue, SIRT1 induced fat release into the bloodstream, and is activated by starvation. In skeletal muscle, SIRT1 is required for PGC-1α deacetylation, and then shift to fatty acid oxidation subsequently during nutrient deprivation. Systemic activation of SIRT1 may be detrimental to some tissues, but beneficial to others, it results in zero net lifespan extension. Much controversy has arisen around overexpression in nematodes and Drosophila, but few attempts have been made to further characterize the tissue-specific roles and define activities of sirtuins in these genetic systems by manipulation—for example, 20 years after the initial studies, no tissue-specific role for nematodes SIR-2.1 has been reported to date. Notably, there are six other sirtuins in mammals. The role of Sirt6 in metabolism and longevity is less well studied but may be important, with one research displaying that Sirt6 overexpression prolongs lifespan in male mice [281].

### Telomerase activators

Telomere length is a marker of cellular aging that shortens with age and is associated with aging-related diseases, including cancer, cardiovascular disease, arthritis, stroke, cataracts, type 2 diabetes, hypertension, osteoporosis, dementia, and chronic obstructive pulmonary disease. Environmental factors, including diet and lifestyle, can affect the rate of telomere decreasing, but telomerase can reverse it. Telomerase is active in highly proliferative cells, such as cancer cells, male germ cells, activated lymphocytes and stem cells. Telomerase activation is considered to be an anti-aging regulator and may be used to treat aging-related diseases [288]. There are many studies linking dietary supplements to telomere length, telomerase activity, and oxidative stress, and it was recognized that natural products with several antioxidants are more effective than taking one antioxidant, implying a synergistic effect between these compounds [289, 290].

Telomere maintenance is an important part of cellular repair. Telomere loss is a marker of aging, because comprehensive work has found that telomeres shorten with each cell division [291]. Cancer cells solve this problem through the overexpression of telomerase to lengthen the ends of chromosomes. Although telomere shortening is associated with aging, the main concerns are the therapeutic value of overexpressing enzymes that prevent telomere depletion because of the potential to cause cancer. However, it has recently been shown that activation of telomerase delays aging and, importantly, in mutant mice, this is achieved without increasing the incidence of cancer [292]. Taken together, these examples evidently demonstrate that preventing DNA damage and maintaining genomic integrity are particularly important in aging. Furthermore, the delicate balance between maintaining the stem cell pool and suppressing tumors should be carefully considered [293].

### mTOR pathway inhibition

The mTOR pathway is a crucial modulator of anabolic processes and a central controller of cell growth. It consists of two distinct multiprotein complexes, including mTORC1 and mTORC2. mTOR signaling pathway has been the focus of aging research [294]. There is evidence that the mTOR signaling pathway acts as a key controller of cellular senescence. Previous studies have shown that mTORC1 has a more prominent role in senescence than mTORC2. Inhibition of the mTOR signaling pathway prolongs lifespan through the simulated effects of dietary restriction, whereas long-term activation of mTORC1 prolongs the age-dependent conditions. In model organisms such as fruit flies and worms, mutation of mTORC1 signaling pathway components or activation of upstream inhibitors prolongs lifespan [295]. Rapamycin can delay the aging process in mammals by inhibiting mTORC1 to improve insulin sensitivity and glucose tolerance [296]. Several genetic and molecular biology studies have shown that diet restriction and mTOR kinase regulate aging with mechanistic overlap [297]. Nutrient richness plays a critical role in the initiation and development of aging. Diet restriction is a natural way to delay aging by inhibiting mTORC1. AMPK can be induced by calorie restriction, which successively activates TSC1-TSC2, thereby inhibiting mTORC1 signaling and prolonging life [298].

In recent years, significant advances have been made in the deeper understanding of pathogenesis in the molecule level and pathways in the cell level involved in aging. It is promising to delay or postpone the onset of diseases associated with aging. Given the economic and social burden of a rapidly growing aging population, the mTOR signaling pathway will have more therapeutic uses in the near future with the discovery of more effective mTOR inhibitors [299].

## Conclusions and outlook

Aging is a systemic, evolutionarily conserved event that affects almost all organisms. Aging is also characterized by multisystem tissue dysfunction and age-related disease conduction. However, aging is malleable, and in many organisms, interventions are available to extend life, improve health and treat disease. These findings are of incomparable importance in biomedicine, since they have the great potential to lead to unprecedented improvements in health.

For many centuries, rejuvenation and youth maintenance have been issues of scientific curiosity. In recent decades, this has accelerated the establishment of an anti-aging industry. This is a controversial area in biomedical research. According to analysis, the economic impact of delayed aging and increased health span in the United States is estimated at $7 trillion over the next 50 years [300].

China’s major health industry (including anti-aging products) has now developed a market size of over $1.3 trillion per year, with an average annual growth rate of over 10%. It is expected that by 2050, the annual size of the health industry will exceed the U.S. market to reach $3.5 trillion. The size of the anti-aging industry will also exceed that of the United States. Thus, it seems clear that the new drug targets’ discovery based on biogerontology represents an incredible opportunity for industries in healthcare and pharmacy.

Performing clinical trials to study the anti-aging potential of conventional drugs is undoubtedly a very difficult task. This is because older patients often suffer from multiple diseases and receive multiple medications simultaneously. The presence of drug–drug interactions and identified comorbidities make the evaluation of such drugs difficult, especially to assess the full range of effects produced by these drugs, whether beneficial or harmful. The lack of reliable and detectable biomarkers to assess the effectiveness of anti-aging interventions is another serious challenge. Initial trials should first be devised to treat diseases and conditions associated with aging, and should be conducted in a small cohort, over relatively short periods of time, with a primary focus on safety and tolerability. After it provides early clues to particularly promising potential candidates, it is then worthwhile to conduct longer or more detailed studies focusing on anti-aging.

The criteria for a potential anti-aging drug are: (1) a drug that extends the lifespan of a model organism, preferably a mammal; (2) a drug that delays or prevents some aging-related diseases in mammals; and (3) a drug that inhibits the senescence transition of cells from quiescence to senescence. The criteria may overlap. If an intervention is intended to extend lifespan, it must retard diseases associated with aging [301].

**Scheme 7:**
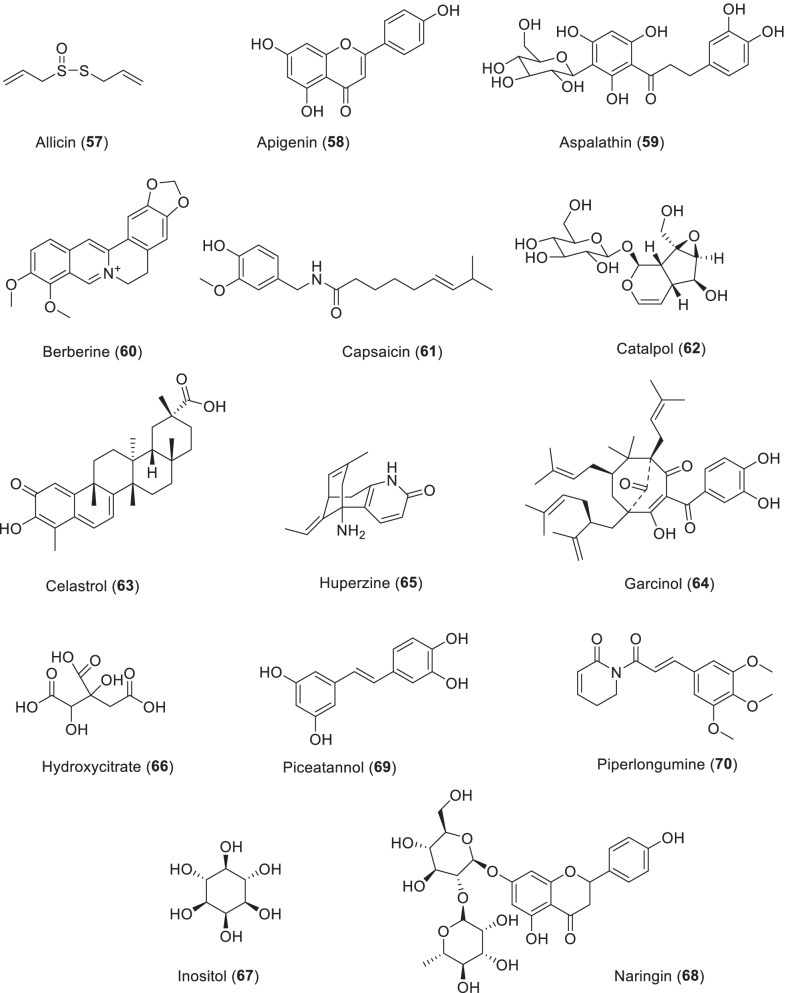
The structures of natural products with antiaging effects

Many plants and fungi as food, beverages, and spices were consumed. They contain natural anti-aging products that can extend the lifespan of model organisms. These active molecules regulate the same cellular and physiological pathways that are affected by calorie restriction (CR) and exercise. Compounds that increase lifespan and healthspan mimic the effects of CR, typically by reducing insulin/IGF-1 signaling and activating autophagy and other cellular processes that increase resistance to stress. These natural products not only increase lifespan, but also improve health and quality of life by reducing the development of chronic diseases, including cancer, diabetes, cardiovascular disease, and neurodegeneration. Various strategies exist for using the anti-aging agents described here, including dietary supplements, increasing the intake of foods containing large amounts of these molecules, and/or consuming probiotics and prebiotics that raise blood levels of these molecules. Several nutrients and natural compounds have been observed to be related to increased lifespan in humans, suggesting that such strategies are feasible for slowing aging and increasing health span. Plant and fungal molecules with anti-aging properties in model organisms may also lead to the discovery and identification of new bioactive compounds for the development of improved CR mimetics to slow human aging. Except for mentioned above natural products, many other compounds have been reported to show anti-aging activity, such as acetic acid, allicin (**57**), apigenin (**58**), aspalathin (**59**), berberine (**60**). Capsaicin (**61**), catalpol (**62**), celastrol (**63**), garcinol (**64**), huperzine (**65**), hydroxycitrate (**66**), inositol (**67**), naringin (**68**), piceatannol (**69**), and piperlongumine (70) (Scheme 7) [302, 303].

*Grifola frondose*, commonly known as Maitake in Japan and Hui-Shu-Hua in China, is an edible mushroom with nutritional and medicinal properties. Polysaccharides are the most important bioactive components of *G. frondose*, contributing to its many biological activities and health benefits, and have been approved for immunotherapy and adjuvant treatment of cancers with chemotherapy and radiotherapy in humans [304]. Zinc-enriched polysaccharides from *G. frondose* have shown anti-aging abilities in vivo [305]. The polysaccharides extracted from other mushrooms, such as *Ganoderma lucidum* and *Agaricus blazei*, were also confirmed to have anti-aging effects [306, 307].

*Astragalus membranaceus* (Huangqi), *Lycium barbarum* (Gouqi), *Rehmannia glutinous* (Dihuang) are major medicinal herbs that have been generally used in many formulations for treatment of a wide variety of diseases and body disorders in the practice of TCM (traditional Chinese medicine), or marketed and used in China as the extracts of life span extension for more than 2000 years. The polysaccharides extracted from these herbs can extend a healthy lifespan in worms or mice [308–310].

Biogerontology is entering a period of exciting and rapid development. It has great potential for future pharmacological interventions to slow aging. As a new era of anti-aging drug discovery dawns, the research community will need to pay special attention to the timely development of drugs that can slow the aging process, either alone or as multiple agents (polypill). Natural products provide the driving force to move forward in our quest to understand and improve the health span, just as they have always done! In regulating aging, it is hoped that these drugs will also reduce the burden of many age-related diseases [300].
